# CRISPR/Cas9 technology in tumor research and drug development application progress and future prospects

**DOI:** 10.3389/fphar.2025.1552741

**Published:** 2025-07-08

**Authors:** Han Han, Xiaoyan Sun, Xiaoyun Guo, Jiaxin Wen, Xiaoming Zhao, Weiqiang Zhou

**Affiliations:** ^1^ Department of Biochemistry and Molecular Biology, School of Basic Medicine, Shenyang Medical College, Shenyang, Liaoning, China; ^2^ Department of Pathogen Biology, School of Basic Medicine, Shenyang Medical College, Shenyang, Liaoning, China

**Keywords:** CRISPR/Cas9 technology, tumor research, gene editing, drug target screening, cancer therapy

## Abstract

The CRISPR/Cas9 system is an acquired immune defense mechanism that has evolved in bacteria and archaea to protect against viral and plasmid attacks. It consists of regularly spaced clusters of short palindromic repeats (CRISPR) and CRISPR-associated proteins (Cas). By adapting the simplest type II CRISPR system to utilize special small guide RNA (sgRNA) and Cas9 nucleic acid endonuclease, precise cuts can be made at specific locations in double-stranded DNA, facilitating gene knockout or knock-in. Due to its efficient gene editing capabilities, CRISPR/Cas9 technology has been widely adopted across various biological and scientific research fields, demonstrating significant potential in tumor research and drug development. This article reviews the progress and future prospects of CRISPR/Cas9 technology in tumor genome editing, drug target screening and validation, and new drug development. It details the fundamental role of this technology in cancer biology research, encompassing various aspects such as gene transcription editors, epigenetic editors, precision genome engineering, and CRISPR-Cas systems targeting RNA. Additionally, the article discusses key applications of CRISPR/Cas9 in anticancer drug discovery, including drug target identification, drug target screening and validation, combinatorial genetic screening, screening of small molecules to overcome resistance to CAR-T therapies, and multimodal functional genomics integration strategies. Finally, although CRISPR/Cas9 has demonstrated great potential for efficient gene editing, precise target discovery, and promotion of personalized therapy and drug screening in oncology research, its application still faces technical bottlenecks such as off-target effects, genomic instability, and low editing efficiency in solid tumors, as well as ethical controversies in gene editing, safety assessment of delivery systems and immune responses in clinical translation, and other ethical and translational challenges.

## 1 Introduction

Tumors are characterized by multiple genotypes, and genetic variants play a crucial role in the development of malignant tumors and their associated chemoresistance. Correcting or deleting these variant genes represents a promising direction for the future development of tumor therapies (Li S. et al., 2024). In recent years, the field of gene editing has advanced rapidly, with genome editing technologies utilizing sequence-specific nucleases (SSNs) sparking a global research boom (Pacesa et al., 2024). In 2012, Science recognized SSNs, particularly those represented by TALEN, as one of the top 10 scientific advances of the year, referring to them as the “genome cruise missile.” The following year, Science again highlighted CRISPR/Cas9, a new star among SSNs, as one of the top 10 scientific advances. Furthermore, in 2014, Nature Methods identified genome editing as one of the 10 most influential research methods in biology over the past decade (Singh et al., 2024).

**FIGURE 1:**
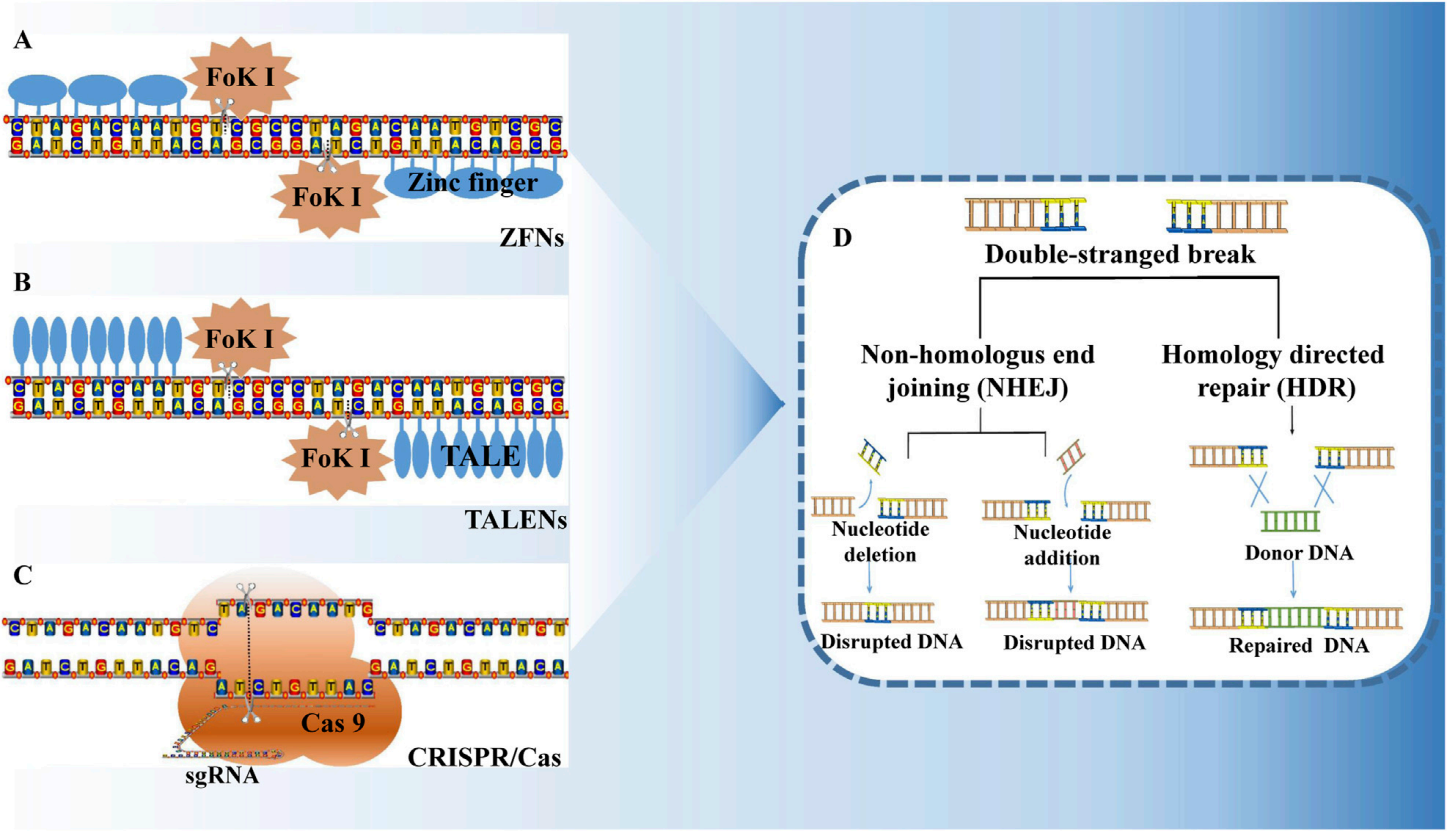
Zinc finger nuclease **(A)**, transcription activator like effector nuclease **(B)**, and CRISPR/Cas system **(C)** are the three major technologies for gene editing. All of them can cause double-strand breaks at specific locations on the DNA, activating the DNA repair mechanism within the cell **(D)**. DNA repair is mainly through two pathways: non-homologous end joining (NHEJ) and homologous recombination (HR). NHEJ repair is imprecise and often leads to gene knockout. HR repair is accurate and can achieve point-specific replacement or insertion of genes.

Zinc finger nucleases (ZFNs), transcription activator-like effector nucleases (TALENs), and CRISPR/Cas (clustered regularly interspaced short palindromic repeats/CRISPR-associated) systems are recognized as among the top 10 scientific advances of the year (Ren et al., 2019). These artificial nucleases have dramatically transformed the methods by which researchers study genes and their functions in mammalian systems. They can induce DNA double-strand breaks (DSBs) at specific DNA target sites, and targeted genome editing is achieved by manipulating the DNA repair pathways (Choudhery et al., 2025). The DSBs generated following DNA damage activate the cell’s intrinsic non-homologous end joining (NHEJ) system. Two distinct repair mechanisms, namely non-homologous end joining (NHEJ) and homologous recombination (HR), are employed to repair the damaged DNA, thereby facilitating targeted genome editing (Anzalone et al., 2020) (Figure 1D). Cells utilizing the NHEJ pathway often exhibit insertions and/or deletions of nucleotide fragments, along with other mutations in their genomes, which complicates precise control over the mutation outcomes. In contrast, the genome sequence of cells undergoing homologous recombination typically remains unchanged, allowing for precise modification and transformation of the genome through the introduction of homologous recombination donor DNA. The NHEJ approach reconstitutes broken chromosomes, albeit often imprecisely, resulting in small insertions or deletions at the break site and leading to knockout mutants. Conversely, the HR approach employs homologous sequences as templates for synthetic repairs, yielding precise substitution or insertion mutants. Among these two pathways, the NHEJ mechanism is predominantly utilized and can occur across nearly all cell types and cell cycle phases (G1, S, and G2), whereas HR is less frequent and primarily occurs during the S and G2 phases (Merker et al., 2024).

## 2 The fundamental role of CRISPR-Cas9 in cancer biology research

ZFNs are formed by the fusion of ZFA, a transcription factor protein that contains a tandem zinc finger DNA domain, and the cleavage domain of Fok Ⅰ, a non-specific nucleic acid endonuclease (Igoucheva et al., 2001) (Figure 1A). The DNA-binding domain of ZFNs specifically recognizes and binds to their corresponding DNA sequences. When the two ZFN monomers are bound to their respective target sites, they form a cleavage-active Fok Ⅰ dimer that cuts the DNA at the target location (Hilioti, 2018). Similarly, TALENs utilize the DNA-binding domain of the TAL effector in conjunction with the non-specific nucleic acid endonuclease Fok Ⅰ (Boch et al., 2009) (Figure 1B). TALENs are synthetic nucleases that incorporate the DNA-binding domain of the TAL effector and the cleavage domain of Fok Ⅰ. TALs consist of multiple tandem repeating units, each comprising 33–35 amino acids. The amino acids within these repeating units are highly conserved, although positions 12 and 13 are variable and are often referred to as the variable regions. The two TAL monomers of TALENs bind to the DNA double strand, forming a Fok Ⅰ dimer with cleavage activity that targets and cuts the DNA at the specified site. TALENs have been extensively studied and applied in genome-targeted modifications (Hu et al., 2024).

TALEN utilizes a single unit of Talen protein to recognize one base as the guide region; particularly TALENs usually require the design of a novel TALE domain for each specific gene to be modified, requiring complex and highly expensive protein engineering (Miller et al., 2011). ZFNs, on the other hand, utilize a single unit of zinc finger protein recognizing three bases to act as a guide chain. ZFNs and TALENs are two technologies that are technically more difficult to implement and take longer to construct and assemble, making it difficult for general laboratories to utilize them effectively.

Compared to ZFNs and TALENs, which use proteins as target recognizers, the CRISPR system’s advantage lies in its unique RNA-guidance mechanism: only about 20 bp of single-stranded guide RNA (sgRNA) needs to be designed to achieve target recognition, completely simplifying the complex protein engineering process of traditional technologies - ZFNs rely on zinc finger protein structural domain design. While TALENs require cumbersome assembly of repetitive amino acid modules. In contrast, ZFNs rely on the targeted design of zinc finger protein structural domains and TALENs require the tedious assembly of repetitive amino acid modules, both of which often take weeks to construct, CRISPR vectors can be prepared in a matter of days (Jinek et al., 2012). This design innovation not only dramatically reduces the technological threshold and experimental cost, but also gives CRISPR excellent multi-gene editing capabilities: by co-delivering multiple sgRNAs, CRISPR can be efficiently multiplexed in mammalian cells, with editing efficiencies of more than 80%, which is significantly better than that of ZFNs and TALENs, which are generally less than 30% efficient (Cong et al., 2013). In addition, the scalability of the CRISPR system is also an important advantage. The integration of inactivated Cas9 (dCas9) and transcriptional regulators (e.g., CRISPRa/CRISPRi technologies) eliminates the need to rely on DNA double-stranded cleavage to dynamically regulate gene expression, thus providing a flexible tool platform for the precise manipulation of complex biological processes. In the process of new drug discovery, efficient target screening and validation is an extremely critical step. The emergence of CRISPR/Cas9 technology realizes the targeted gene editing, which makes it more convenient to construct animal models or cell line models, and greatly accelerates the screening and validation of oncology drug targets as well as the research and development of new drugs.

In the field of targeted cancer therapy, CRISPR/Cas9 technology shows great potential for application. On one hand, CRISPR/Cas9 can be used to target oncogenic gene destruction, such as CRISPR-LNP (lipid nanoparticle) delivery system can deliver Cas9 mRNA to glioblastoma cells to specifically knock down oncogenic genes (e.g., EGFRvIII), which significantly extends the survival period of mice; on the other hand, in the personalized immunotherapy, CRISPR editing of the PD-1 gene of T cells can enhance the effect of CAR-T cells on tumor cells. Enhance the killing effect of CAR-T cells on solid tumors (Eyquem et al., 2017; Rupp et al., 2017), and recent studies have also demonstrated that the activation of tumor antigen expression by the CRISPR system controlled by focused ultrasound (FUS) can significantly improve the infiltration efficiency of CAR-T cells *in vivo*. In addition, CRISPR/Cas9 technology can also be used to repair or knock out oncogenes, and activate or inhibit oncogenes, such as repairing mutations in the tumor suppressor gene TP53, which can restore its normal function and thus inhibit the growth of tumor cells. Meanwhile, this technology also plays an important role in constructing cancer models and screening cancer therapeutic targets, providing a key aid to cancer research and treatment. In terms of clinical translation, Casgevy, the world’s first approved CRISPR drug, provides a powerful reference for cancer gene therapy.

### 2.1 Overview of CRISPR-Cas9 technology

The CRISPR/Cas system is a natural immune mechanism commonly found in bacteria and archaea, primarily functioning to resist invading viruses and exogenous DNA. This system comprises the CRISPR sequence and the Cas gene family. The CRISPR sequence consists of a series of highly conserved repeats interspersed with unique spacers, while the Cas genes encode proteins with nuclease functions capable of specifically cleaving DNA sequences (Pan and Zhang, 2021). The Cas genes can be categorized into type I, type II, and type III systems based on the sequence of the core element of the Cas gene, with type II systems requiring only a single Cas9 protein to achieve DNA cleavage (Li Z. et al., 2024).

The CRISPR/Cas9 system encompasses three key components: CRISPR RNA (crRNA), trans-activating crRNA (tracrRNA), and Cas9 endonuclease (Allemailem, 2024). A portion of the crRNA sequence can bind to tracrRNA through base complementary pairing, forming a chimeric RNA (tracrRNA/crRNA). One segment of the crRNA sequence binds to the tracrRNA via base complementary pairing, while the other segment binds to the target DNA site (Chen et al., 2017) (Figure 1C). This chimeric RNA recognizes a specific NGG sequence known as the Protospacer Adjacent Motif (PAM) site, directing the Cas protein complex to bind to this specific site to cleave the DNA double strand (Rosello et al., 2023).

The Cas9 protein contains a RuvC-like domain at the amino-terminal end and a HNH nuclease domain in the middle of the protein. The HNH nuclease domain cleaves the template strand that is complementary to the sgRNA, and the RuvC-like domain cleaves the other strand. The cleavage site is located 3 nt upstream of PAM. Since 2012, the type II CRISPR/Cas system has been optimized to use Cas9 protein and sgRNA to form a simple sgRNA/Cas9 system, which can perform targeted DNA cleavage in eukaryotes similar to ZFN and TALEN (Zuo and Liu, 2017).

### 2.2 Engineering and application of CRISPR-Cas9

In the artificial construction of the CRISPR/Cas9 system, crRNA and tracrRNA sequences are fused to form a single guide RNA (sgRNA), which is then combined with Cas9 to create a complex. This engineered CRISPR/Cas9 is referred to as RNA-guided endonucleases (RGENs), capable of targeting either a single gene or multiple genes, both of which allow for effective site-specific editing (Nujoom et al., 2024). Among these components, the Cas9 protein functions as a nuclease to cleave double-stranded DNA, while the sgRNA determines the specificity of the target sequence through base complementary pairing. Building on this discovery, a new gene editing system that requires only two elements, sgRNA and Cas9, has been successfully developed for gene editing in mammalian cells (Allemailem, 2024).

The simplicity and modularity of CRISPR/Cas9 make it an ideal tool for genome engineering. A particularly notable feature of CRISPR/Cas9 is its modular design; since the targeting module (sgRNA) and the nucleic acid endonuclease module (Cas9) are encoded separately, each module can be modified, evolved, and optimized independently without affecting the function of the other. Furthermore, the short protospacer adjacent motif (PAM) requirement allows for the targeting of virtually any genomic locus using CRISPR/Cas9 (Allemailem, 2024).

In February 2013, a team led by Lei Qi at Stanford University discovered a specific variant of *Streptococcus* pyogenes Cas9 (spCas9) that lacks endonuclease activity, referred to as dead Cas9 (dCas9). When co-expressed with guide RNA (gRNA), this dCas9 forms a DNA recognition complex that binds to specific DNA target sequences (Kovalev et al., 2024). This binding prevents the interaction of RNA polymerase or transcription factors, thereby interfering with the process of transcription elongation. This discovery expands the function of Cas9 from its original role as an RNA-guided nuclease to a new domain involving RNA-guided nucleic acid-binding proteins. Furthermore, dCas9, as a derivative of genome editing technology, can be fused with various functional proteins to facilitate a range of targeted modifications, including transcriptional activation, transcriptional repression, epigenetic regulation, and targeted RNA editing. Notable functional proteins reported in this context include VP64, KRAB, and TET1 (Chavez et al., 2023; Becirovic, 2022; Peddle et al., 2020; Jogam et al., 2022).

#### 2.2.1 Gene transcription editor based on CRISPR-Cas9 system

Building on the unique properties of dCas9, Lei Qi’s team developed two innovative tools for gene expression regulation: CRISPRa (CRISPR activation) and CRISPRi (CRISPR interference). CRISPRa activates gene expression by fusing dCas9 with a specific transcriptional activator, enabling it to target and bind to a specific DNA sequence (Schmidt et al., 2022). Conversely, CRISPRi employs dCas9 fused to a transcriptional repressor, which similarly targets and binds to a specific DNA sequence to inhibit gene expression in its vicinity (Ilyas et al., 2024).

These tools offer unprecedented precision and flexibility in gene regulation, empowering scientists to study and manipulate genes at a more granular level within cells. The advent of CRISPRa and CRISPRi has undeniably transformed the fields of gene therapy, gene editing, and biological research (Razavi et al., 2024).

##### 2.2.1.1 CRISPRi system

**FIGURE 2:**
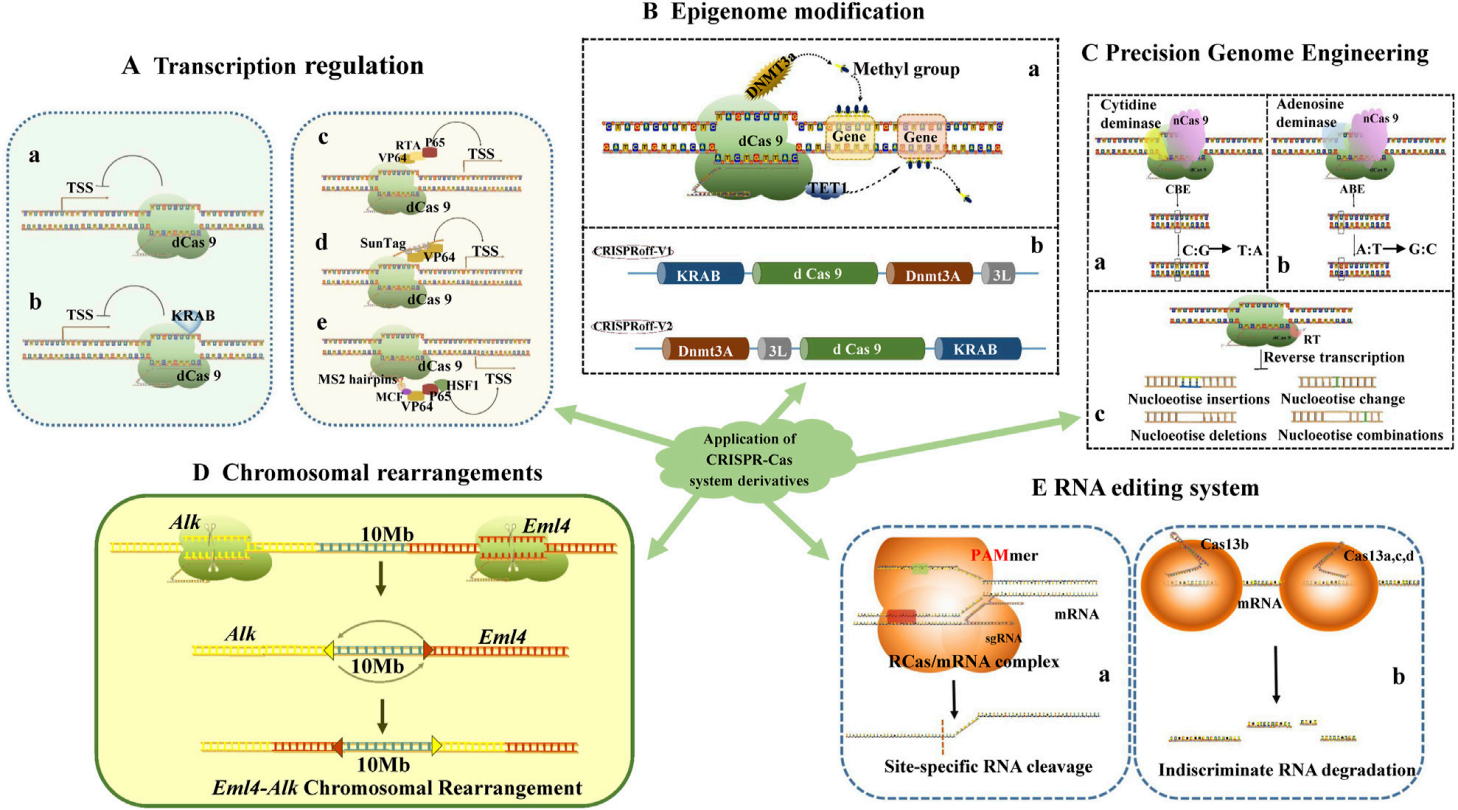
Application of CRISPR-Cas system derivatives: Transcription regulation **(A)**, Epigenome modification **(B)**, Precision Genome Engineering **(C)**, Chromosomal rearrangements **(D)**, RNA editing system **(E)**.

In *E. coli*, CRISPRi technology employs a specialized sgRNA that comprises a gene-targeting sequence, a Cas9 protein binding sequence, and a transcription termination sequence. When dCas9 binds to this sgRNA, the resulting complex effectively obstructs RNA polymerase from transcribing the target gene sequence in a spatially site-blocked manner, thereby directly inhibiting the extension of gene transcription. This process does not necessitate the degradation of the mRNA produced during transcription and differs from the gene-silencing mechanism of RNA interference (RNAi) (Ilyas et al., 2024). CRISPRi inhibits the transcription of target genes through two primary mechanisms (Konermann et al., 2015) (Figure 2A): first, it prevents RNA polymerase from binding to the promoter of the target gene, thereby inhibiting the initiation of transcription; second, it binds to the open reading frame of the target gene to inhibit the extension of transcription, functioning similarly to a transcription terminator (Alexander et al., 2024).

However, in mammalian cells, the gene expression inhibition effect of the aforementioned CRISPRi complexes is relatively weak. To enhance the efficacy of transcriptional repression, researchers have expressed dCas9 fused with various transcriptional repressors, discovering that the dCas9-KRAB fusion protein exhibits a higher repression efficiency.

The KRAB (Krüppel-associated box) structural domain is a highly conserved element found in zinc finger proteins (ZNFs), typically spanning approximately 75 amino acids and capable of forming two amphipathic α-helices. The KRAB structural domain derived from the KOX1 (ZNF10) protein, which has also been utilized for TALE-mediated gene expression repression (Alerasool et al., 2020), is frequently employed in CRISPRi studies (Konermann et al., 2015) (Figure 2A).

The KRAB structural domain can bind to the co-repressor protein KAP1 (KRAB-associated protein 1), which subsequently recruits the histone methyltransferase SETDB1 (SET domain bifurcated histone lysine methyltransferase 1) (Oleksiewicz et al., 2024). SETDB1 promotes the trimethylation of histone H3K9 (H3 lysine 9), a crucial marker for transcriptional repression that leads to the silencing of target gene transcription (Prashanth et al., 2024). Recent studies have revealed that ZIM3 KRAB demonstrates a more pronounced role in gene expression repression compared to KOX1 KRAB. This finding offers novel insights for further optimizing the CRISPR interference (CRISPRi) system and enhancing the efficiency of transcriptional repression. With ongoing research and refinement, CRISPRi technology is anticipated to assume an increasingly significant role in gene function research, disease treatment, and drug discovery (Ilyas et al., 2024).

##### 2.2.1.2 CRISPRa systems

By linking dCas9 to various regulatory factors, CRISPRi and CRISPRa systems can be developed to modulate gene expression. In the CRISPRi system, dCas9 is associated with transcriptional repressor regulators to inhibit the expression of targeted genes. Conversely, linking dCas9 to transcriptional activation regulators leads to the formation of the CRISPRa system, which facilitates gene transcription activation (Cai et al., 2023).

Current CRISPRa systems can be classified into three distinct categories. The first category involves the construction of fusion proteins by linking one or more transcriptional activators in tandem with dCas9. For instance, fusing the C-terminus of dCas9 with the activation domain VP64, which comprises four VP16 structural domains, enhances transcriptional upregulation when stably expressed in HEK293T cells (Becirovic, 2022). Additionally, multiple homologous or heterologous transcriptional activators, such as dCas9-VPR (VP64-p65-Rta), can be fused in tandem, resulting in significantly improved activation (Lim et al., 2020; Chavez et al., 2015) (Figure 2A).

The second category of CRISPRa systems involves the creation of fusion proteins that combine dCas9 with scaffolding proteins to recruit multiple transcriptional activators (Kanafi and Tavallaei, 2022). An example of this is the use of peptide array scaffolds that specifically bind to transcriptional activator fusion proteins through tandem fusion, such as SunTag (Ding et al., 2022; Cheng et al., 2013) (Figure 2A).

A third class of CRISPRa systems involves the simultaneous construction of transcription activator fusion proteins with dCas9 and sgRNAs that contain MS2 aptamer sequences for recruiting transcription activator complexes fused to MCP (MS2 coat protein) (Heidersbach et al., 2023). This design facilitates the concurrent binding of multiple transcriptional activators, thereby enhancing the transcriptional activation effect. In such systems, sgRNAs comprise both prototypical sgRNAs and engineered sgRNAs capable of binding to specific protein tags (Dong et al., 2022). For example, the CRISPR-SAM system (synergistic activation mediator) includes a dCas9-transcriptional activator fusion protein, a nucleic acid aptamer sequence containing the MS2 sequence that binds to the MCP sgRNA, and an MCP-transcriptional activator fusion protein, which can specifically activate most intracellular genes (Feser et al., 2023). In the SAM system, sgRNA incorporates an MCP-binding aptamer sequence that interacts with the ternary fusion proteins of MCP, p65, and HSF1 to enhance transcriptional activation. Furthermore, the transcriptional activation effect of single-site sgRNA-directed SAM in the promoter region is significantly greater than that of multisite dCas9-VP64 (Zhang et al., 2019; Omachi and Miner, 2022; Komor et al., 2017) (Figure 2A).

In summary, the CRISPRa system achieves efficient activation of gene transcription through various dCas9 fusion proteins and sgRNA constructs, providing a powerful tool for gene therapy, gene editing, and related fields. Although CRISPRa and CRISPRi technologies have the advantage of precisely regulating gene expression, they still face many challenges in practical applications. In heterochromatin regions, the highly compressed chromatin structure prevents the binding of dCas9-effector complexes to target DNA, resulting in lower regulatory efficiency, e.g., less than 20% of gene activation efficiency in H3K27me3-tagged regions in hepatocellular carcinoma. In addition, effector proteins such as VP64 and KRAB are difficult to recruit in heterochromatin, which also affects the regulatory effect. For *in vivo* applications, large-sized effector complexes (e.g., dCas9-VPR) are limited by the capacity constraints of AAV vectors (∼4.7 kb), which makes it difficult to achieve efficient co-delivery (Nyberg et al., 2023); whereas long-term expression of viral vectors, such as lentiviruses, may trigger immune responses against Cas9 or effector proteins (Charlesworth et al., 2019). Non-viral vectors such as lipid nanoparticles (LNPs) can circumvent the immune risk, but *in vivo* stability and tissue targeting still need to be optimized. Non-specific binding of sgRNAs and competitive binding due to the sharing of the dCas9 backbone by the CRISPRa/CRISPRi system may also trigger signal crosstalk and off-target regulation (Zhang et al., 2015).

To overcome these limitations, the following strategies can be used: at the epigenetic remodeling level, by fusing dCas9 with chromatin-opening factors (e.g. p300 histone acetyltransferase), sites such as H3K27 can be locally acetylated to directly remodel heterochromatin structure. For example, the dCas9-p300 system promotes chromatin opening, resulting in a 3.8-fold increase in gene activation efficiency compared to traditional methods (Huang et al., 2023); strategies targeting methylases or demethylases (e.g., dCas9-TET1, dCas9-LSD1) can also improve target access by reversibly modifying the DNA methylation status (Brocken et al., 2018). Combining MNase-seq data to design sgRNAs targeting vacant regions of nucleosomes or optimizing sgRNA sequences to improve binding affinity (Doench et al., 2016) enhances the regulatory efficiency of heterochromatin regions at the level of target selection.

In terms of *in vivo* delivery and safety, the dual AAV vector system packages dCas9 separately from effector proteins (Zhi et al., 2022; Yedier-Bayram et al., 2022), which effectively solves the capacity limitation of a single vector, and the transient expression scheme of LNP-delivered Cas9 mRNAs and sgRNAs (Zong et al., 2023), which significantly reduces immunogenicity. Delivery efficiency and safety are further balanced by engineering modified effector proteins (e.g., miniature KRAB structural domains, SunTag signal amplification system) or employing non-immunogenic Cas9 homologs (e.g., SaCas9) and humanized modifications (Lebek et al., 2024). The introduction of spatiotemporal-specific regulatory technologies (e.g., photocontrol systems or rapamycin-inducible systems) not only realizes precise spatiotemporal regulation of gene expression (Iqbal et al., 2023), but also reduces the potential risks associated with sustained expression. In addition, targeted delivery systems have significantly enhanced tissue targeting by modifying LNP surface ligands (e.g., tumor-specific antibodies) or engineering modified AAV coat proteins, providing viable options for organ-specific gene regulation in specific organs such as the liver and central nervous system.

The synergistic application of these strategies not only targets the structural barriers of heterochromatin regions but also systematically optimizes the precision of *in vivo* delivery and regulation, laying a key foundation for the translation of CRISPRa/CRISPRi technology from basic research to the clinic. Future research could further integrate the innovation of epigenome mapping and delivery systems to promote the precise application of this technology in gene therapy and disease modeling.

#### 2.2.2 Epigenetic editors

Many epigenetic modifiers fused to dCas9 proteins can regulate gene expression by inducing chemical modifications at the DNA or chromatin level, including DNA-targeted methylation and epigenetic modifications such as the targeted acetylation and/or methylation of histones, which are both long-lasting and heritable (Yen et al., 2016). A common strategy for constructing dCas9 epigenetic editing systems involves fusing dCas9 with epigenetic modifying enzymes and leveraging dCas9’s ability to bind to specific DNA targets to achieve epigenetic editing at genome-specific loci (Polstein and Gersbach, 2015) (Figure 2B).

For instance, combining the DNA methylation structural domain of DNMT3a, an active DNA methyltransferase capable of methylating CpG sites *in vivo*, with dCas9 facilitates transient methylation of DNA at the targeted promoter, thereby enabling long-term gene silencing (Stepper et al., 2017). This approach has been applied to SNCA-carrying human iPSC-derived dopaminergic neurons by targeting SNCA intron 1 with dCas9-DNMT3a fusion proteins, resulting in DNA hypermethylation and providing evidence from *in vitro* studies for potential Parkinson’s disease treatments (Kim et al., 2023).

Conversely, the fusion of the catalytic structural domain of methylcytosine dioxygenase 1 (TET1) with dCas9 allows for rapid demethylation of the promoter, leading to the upregulation of target gene expression and driving transcriptional activation in various cell types. dCas9-TET1 fusion proteins thus hold significant therapeutic potential for treating diseases such as cancer and renal fibrosis (Xu et al., 2023). Additionally, the immune response can also be modified with this strategy. This has been shown in human and mouse lymphocytes, where targeted demethylation of master regulators can be part of a strategy to favor certain lineages, such as the FOXP3-TSDR for Tregs (Kressler et al., 2021; Wilk et al., 2022; Aroyo-Olarte et al., 2024).

In addition to the methylation modification of DNA, histone modification can also achieve similar gene regulatory effects. For instance, the fusion of the p300 catalytic structural domain with dCas9 enables transient and efficient acetylation of histones when targeting enhancer and promoter regions, thereby enhancing gene expression. Conversely, when dCas9 is fused with HDAC1, it removes acetylation and inhibits cancer growth (Hilton et al., 2015).

Recently, a novel CRISPR-based epigenetic editing technology, termed CRISPR off, has been reported. Researchers fused KRAB and D3A-D3L to the N- and C-terminus of dCas9, respectively, leading to the development of new epigenetic editors CRISPRoff-V1 and CRISPRoff-V2 (Nuñez et al., 2021) (Figure 2B). It was observed that CRISPR off, particularly CRISPRoff-V2, could durably inhibit the expression of GFP reporter genes, with its short-lived expression of transcriptionally inhibitory effects lasting for at least 50 days. Subsequently, the researchers also designed CRISPR on, which effectively reverses CRISPR off-mediated DNA methylation modification and transcriptional repression (Nuñez et al., 2021). The CRISPR off/on system thus provides a powerful tool for controlling gene expression, targeting enhancers, and exploring the principles of epigenetic inheritance. In general, the altered epigenetic, landscape of tumor cells can be restored with these tools. For example, dCas9-DNMT3a to block oncogenesand dCas9-TET1 to recover tumor repressor gene expression (Neja, 2020).

#### 2.2.3 Precision genome engineering

Another key application of CRISPR/Cas9 is precision genome engineering. By supplying cells with a DNA template for homology-directed repairs (HDRs) following a Cas9 cut, nearly all forms of sequence alterations can be precisely engineered. These alterations include point mutations, small insertions and deletions, and large deletions. In the context of cancer biology, this approach is particularly valuable for constructing cell lines and animal models that possess complex genetic alterations, thereby mimicking the mutational profiles observed in human tumors.

##### 2.2.3.1 Base editors

In mammalian cells, the CRISPR-Cas system facilitates DNA sequence alterations by precisely cleaving target genes to create double-strand breaks (DSBs) and subsequently relying on the intracellular DNA repair mechanisms, namely homologous recombination (HR) and non-homologous end joining (NHEJ), to mend these breaks. While HR can achieve precise and controllable editing, its efficiency remains relatively low. Conversely, NHEJ, although more efficient, may introduce insertion/deletion mutations (indels) during the repair process, which hampers precise gene editing and increases the likelihood of side effects associated with large-scale DSBs. Consequently, for many pathogenic genetic variants caused by single nucleotide variants (SNVs), effective treatment cannot be accomplished through simple gene knockout and knock-in strategies alone; thus, there is a pressing need for the development of methods and tools capable of efficiently correcting SNVs.

To address this requirement, CRISPR-Cas9-based base editors and lead editors have been introduced. These innovative tools do not rely on the DNA cleavage associated with DSBs, significantly mitigating the toxicity linked to erroneous repair and enabling broader applications of the CRISPR-Cas system. Among these, base editors (BEs) represent a class of tools that facilitate efficient and precise editing of specific base types at the single-base level without necessitating DNA cleavage to generate DSBs or homologous donor templates. Instead, they combine programmable DNA-binding proteins with base deaminases to create fusion proteins. The two primary types of DNA base editors currently in widespread use are cytosine base editors (CBEs) and adenine base editors (ABEs), which enable C>T and A>G transitions, respectively (Lam et al., 2023; Liu et al., 2023a).

The core element of Cytosine Base Editing (CBE) is a fusion protein composed of either dCas9 or nCas9 (Cas9 proteins with single-strand cleavage activity) and cytosine deaminase. Guided by single-guide RNA (sgRNA), this fusion protein can locate the target sequence, bind to the sgRNA-unpaired single-stranded DNA (ssDNA), and deaminate the cytosine into uracil. This uracil is subsequently converted into thymine during DNA replication or repair, thereby facilitating the direct substitution of cytosine-guanine (C-G) base pairs with thymine-adenine (T-A) base pairs (Rees and Liu, 2018) (Figure 2C). For instance, in the G93A-SOD1 amyotrophic lateral sclerosis mouse model, the intronic peptide SpCas9-CBE fusion protein, delivered via adeno-associated virus (AAV), successfully edited a specific codon in the SOD1 gene sequence into a termination codon. This resulted in reduced G93A-SOD1 expression, diminished muscle atrophy, and enhanced neuromuscular function (Duan et al., 2020).

Similarly, the core element of Adenine Base Editing (ABE) consists of a fusion protein formed from nCas9 and an artificially evolved adenine deaminase. Guided by sgRNA, this fusion protein can target a specific DNA sequence and deaminate a range of adenines into inosine after Cas9 binds to the target sequence, forming an R-loop. The inosine is then read and replicated as guanine, resulting in the substitution of adenine-thymine (A-T) base pairs with guanine-cytosine (G-C) base pairs (Gaudelli et al., 2018) (Figure 2C). ABE has been utilized in hematopoietic stem and progenitor cells (HSPCs) from patients with sickle cell disease to convert the pathogenic gene HBBS into the non-pathogenic HBBG, a transformation that is durable and minimizes the adverse consequences associated with double-strand breaks (DSB) (Newby et al., 2021). Additionally, ABE has shown promise in treating Hutchinson-Gilford premature senescence syndrome (HGPS) by reversing vascular pathology, maintaining vascular smooth muscle cell numbers, and preventing epicardial fibrosis through the correction of the pathogenic mutation 1824 C>T in the LMNA gene in a mouse model (Lin et al., 2021). These findings underscore the significant potential of ABE for the treatment of genetic disorders.

CBE and ABE are important derivatives of the CRISPR-Cas9 system, capable of realizing C-to-T and A-to-G base conversions without introducing double-stranded DNA breaks (Wang et al., 2024). However, these two editors are limited in their ability to realize only four base conversions (C-to-T, T-to-C, A-to-G, G-to-A) and cannot cover all 12 possible base interchanges. In addition, off-target effects may also occur for non-target bases within the editing window of the base editor, which limits its application in precision gene editing, especially in cancer therapy, where off-target effects may lead to genomic instability or carcinogenic risk.

##### 2.2.3.2 PE editors

To enable the full interchange of the 12 bases, David Liu’s team developed the prime editor (PE) (Doman et al., 2023). PE introduces two key improvements over CRISPR-Cas9: first, a reverse transcriptase RNA primer with a gene-editing sequence is appended to the 3′end of the single-stranded guide RNA (sgRNA) to form the engineered guide RNA (pegRNA), which encompasses both the sgRNA and a primer binding site (PBS) along with the reverse transcription template; second, dCas9 (inactivated Cas9) is fused with reverse transcriptase, allowing dCas9 to cleave the DNA single strand under the guidance of the sgRNA sequence within the pegRNA. The PBS at the 3′end of the pegRNA recognizes and pairs with complementary sequences preceding the cut site. Subsequently, reverse transcriptase utilizes the template sequences following the PBS on the pegRNA for reverse transcription, directly polymerizing the target sequences into the DNA strand at the cut site. This mechanism facilitates the insertion, knockdown, and substitution of small fragment sequences while significantly reducing the off-targeting rate. The robust editing capabilities of PE present substantial therapeutic potential; for example, in a mouse model of Alpha-1 antitrypsin deficiency (AATD), PE successfully eliminated the pathogenic E342K mutation in SERPINA1 by executing A-to-G edits (Erion et al., 2025).

PE provide powerful tools for cancer research with their precise gene editing capabilities (Xu et al., 2023). For example, PE can accurately repair mutations in driver genes (e.g., TP53, KRAS, and EGFR) to restore their normal functions; restore the cancer-suppressive functions of tumor suppressor genes (e.g., PTEN or BRCA1) by inserting or repairing their mutated sites; edit immune checkpoint genes (e.g., PD-1 or CTLA-4) to enhance the tumor-killing ability of T cells; and introduce specific oncogenic mutations in cell or animal models to introduce specific oncogenic mutations for studying the mechanisms of carcinogenesis and drug screening. The advantage of PE over CRISPRa (activation) and CRISPRi (inhibition) is that it enables precise gene editing, not just gene expression regulation. However, CRISPRa and CRISPRi also have unique applications in suppressing tumor growth by activating oncogenes or inhibiting oncogene expression. Therefore, PE, CRISPRa and CRISPRi can complement each other and jointly promote the development of precision cancer treatment (Yang and Zhang, 2023).

Despite the significant advantages of PE in precision editing, it still faces some challenges in practical application. First, the delivery efficiency and editing efficiency of PE still need to be further improved, especially in certain cell types (e.g., primary cells or stem cells), where the editing efficiency is low. Second, the long-term safety of PE still needs to be comprehensively evaluated, especially in in vivo applications, where how to avoid off-target effects and genomic instability remains a key issue (Kantor et al., 2020). In addition, base editors (e.g., CBE and ABE), although capable of specific base conversion, have limited editing windows and cannot cover all 12 base interchanges, and off-target effects may also occur for non-target bases within the editing window.

In the future, PE is expected to play a greater role in cancer therapy as the technology continues to be optimized (Johnsen et al., 2017). By improving reverse transcriptase activity or optimizing pegRNA design, the editing efficiency of PE can be improved, especially in difficult-to-edit cell types (Nguyen et al., 2024). Meanwhile, the combination of single-cell sequencing technology can accurately assess the editing effect, identify the key factors affecting the editing efficiency, and further optimize the editing strategy. In addition, the development of more efficient delivery systems (e.g., AAV or lipid nanoparticles) will help improve the delivery efficiency and long-term stability of PE *in vivo* (Davis et al., 2024). Integrating genomic, transcriptomic and proteomic multi-omics analysis can comprehensively analyze the cellular functional changes after PE editing and provide more comprehensive data support for precision medicine. In conclusion, by continuously optimizing PE technology and combining it with other CRISPR tools (e.g., CRISPRa and CRISPRi), researchers are able to more accurately regulate gene function, promote the development of precision in cancer treatment, and provide more effective treatment options for patients.

#### 2.2.4 Chromosomal rearrangements

Although base editors (e.g., CBE and ABE) and pilot editors (PE) have demonstrated excellent precision in single-base or small fragment editing, the applications of the CRISPR/Cas9 system go far beyond that. Its versatility in genome engineering is also demonstrated in the modification of large-scale genome structures, especially chromosomal rearrangement (CHR). Chromosomal rearrangement, a phenomenon characterized by variation in chromosome structure, involves alterations in the positioning of chromosome segments. Specific forms of these rearrangements include chromosome deletions, duplications, inversions, and ectopic insertions. Such mutations can disrupt normal gene expression and may also induce cellular transformations leading to cancer, which can result in serious diseases such as human lymphoma and leukemia.

Traditional research approaches for creating cancer models involving chromosomal rearrangements often rely on complex genetic engineering techniques. This typically requires the introduction of loxP sequences at two predetermined rearrangement sites, followed by chromosomal rearrangement through the Cre-loxP recombination system. However, this method is cumbersome and relatively inefficient. In recent years, the advent of CRISPR/Cas9 technology has revolutionized the study of chromosomal rearrangement. This innovative technology facilitates chromosomal rearrangement by precisely targeting and cleaving specific DNA sequences. Unlike the traditional loxP-Cre method, CRISPR/Cas9 does not necessitate the introduction of additional sequences and can induce chromosomal rearrangements by targeting only two rearrangement sites for cleavage.

Rearrangements of human chromosome 2 lead to the expression of the EML4-ALK fusion, which is implicated in non-small cell lung cancer. The EML4 and ALK genes are located at bands p21 and p23, respectively, on human chromosome 2, separated by a distance of approximately 10 Mb. The inverted fusion of these gene fragments allows for the expression of the novel fusion protein, EML4-ALK, in tissues. Some researchers have employed virus-mediated CRISPR/Cas9 technology to edit and rearrange adult mouse somatic cells, enabling the rapid establishment of an EML4-ALK fusion gene lung cancer mouse model (Lei et al., 2022) (Figure 2D).

In addition, chromosomal rearrangements are prevalent in acute myeloid leukemia (AML). For instance, AML with MECOM rearrangements represents a rare and aggressive subtype, characterized by alterations in the MECOM gene located on chromosome 3. These rearrangements typically involve translocations, such as inv f (3) or t (3; 3) (Ottema et al., 2020). Similarly, Ewing’s sarcoma is another tumor associated with chromosomal rearrangements, distinguished by a genetic rearrangement of the EWSR1 gene on chromosome 22q12 (Tsuda et al., 2020).

These models provide an effective platform for scientists to investigate the mechanisms underlying cancer development and to screen anti-tumor drugs at the animal level. Through these research advancements, the potential applications of CRISPR/Cas9 technology in cancer research and treatment become increasingly evident.

#### 2.2.5 CRISPR-Cas systems targeting RNA (RCas)

Additionally, researchers have developed RNA-targeted CRISPR-Cas systems (RCas) to circumvent the permanent alterations associated with DNA gene editing and the irreversibility of off-target effects. RCas systems can be broadly categorized into two main types: those that adapt traditional DNA-targeted CRISPR systems for RNA-targeted editing, and those that specifically recognize single-stranded RNA (ssRNA) CRISPR systems. The most representative CRISPR systems include the Cas9 and Cas13 systems within the CRISPR class II. The Cas9 system achieves targeted recognition and cleavage of RNA by incorporating complementary trans-DNA oligonucleotides (PAMmer) that contain protospacer adjacent motifs (PAM) or by engineering PAM-independent dCas9 proteins (e.g., RCas9) (Figure 2E) (O'Connell et al., 2014). Conversely, the Cas13 system specifically recognizes and targets ssRNAs, although its application in gene knockdown is somewhat limited (Li S. et al., 2024) (Figure 2E). Moreover, there are RNA editing technologies that utilize fusion proteins, such as those combining SNAP-tag with base editing enzymes like ADAR or APOBEC1, as well as fusion proteins that recruit base editing enzymes via MS2 aptamers attached to gRNA (Vogel et al., 2018; Bhakta et al., 2020; Haimovich et al., 2016).

Unlike DNA editing systems that rely on a single sgRNA guide, advanced RNA editing systems often employ multiple recognition mechanisms to ensure target specificity. For example, RNA-edited Cas proteins (e.g., Cas13) have RNA recognition capability on their own, and when combined with a specific aptamer system, a dual recognition mechanism can be formed. The REPAIR (RNA Editing for Programmable A to I Replacement) system combines catalytically inactivated Cas13 (dCas13) with the system fuses catalytically inactivated Cas13 (dCas13) with ADAR deaminase, which retains the RNA-targeting ability of Cas13 and utilizes the specificity of ADAR for double-stranded RNA to achieve high-precision editing. It has been shown that this dual recognition mechanism reduces the off-target rate to less than 1/10 of that of conventional DNA editing systems (Cox et al., 2017). The SNAP-ADAR system utilizes chemically induced dimerization to recruit ADAR to the target site, which relies on the natural specificity of endogenous ADAR and reduces unintended editing (Merkle et al., 2019). In addition, strategies such as optimizing the length of fusion protein junctions and introducing high-fidelity deaminase variants can further narrow the editing window and pinpoint the target RNA (Tan, 2023).

The most significant advantage of this system over the conventional CRISPR system is its safety; DNA double-strand breaks can trigger a p53-mediated DNA damage response, leading to cell cycle arrest or apoptosis, especially in primary cells. In contrast, RNA editing does not alter genomic DNA, avoiding these risks. Preclinical studies have shown that RNA editing systems are significantly less cytotoxic than DNA editing systems and can be safely applied in sensitive primary T cells and hematopoietic stem cells.

Second, the dynamic reversibility of RNA editing makes its editing effect fade with RNA metabolism, which is suitable for therapeutic scenarios such as inflammation and metabolic diseases that require dynamic regulation; furthermore, single fusion proteins can target multiple RNAs at the same time through crRNA arrays, which is easier than CRISPR-Cas9, which requires multiple gRNAs and Cas9 proteins (Tong et al., 2023), and Cas13 targets RNAs, which is a more efficient way to target RNAs. ADAR-mediated A-to-I editing can repair RNA errors in diseases such as cystic fibrosis. In addition, gene expression can be regulated by editing RNA modifications (e.g., m6A) to avoid the irreversible risks of DNA editing. Finally, the technology can target non-coding RNAs (e.g. miRNAs, lncRNAs) and regulate gene expression by editing RNA modifications, providing a broader range of intervention strategies for the treatment of complex diseases such as cancer. These properties have been validated in studies such as mRNA repair in spinal muscular atrophy and miRNA-21 targeted editing in breast cancer.

### 2.3 CRISPR-Cas9 experimental models for the discovery and development of anticancer drugs

To address the numerous challenges currently facing the clinic, CRISPR technology has emerged as a pivotal tool in the field of oncology drug development. Many studies have utilized this advanced technology to construct precise cellular, organoid, and animal cancer models. These models not only serve as powerful instruments for the in-depth exploration of the complex molecular mechanisms underlying tumorigenesis and progression, but they also significantly enhance the screening of drug-resistant cell lines and facilitate genome-wide screenings of genes associated with tumor drug resistance. By employing CRISPR technology, we can gain clearer insights into the biological characteristics of tumors, thus establishing a solid foundation for the development of effective and targeted tumor drug therapies.

#### 2.3.1 Cell models

Cellular models are favored in biomedical research due to their short experimental duration, clear backgrounds, and the ease with which results can be obtained. Significant progress has been made using CRISPR-Cas9 technology for two important tumor types: adult neuroblastoma and osteosarcoma. George et al. generated adult neuroblastoma cell lines with ATRX mutations through this technology, which not only facilitated a thorough investigation into the relationship between poor prognosis and ATRX mutations but also enabled the design of innovative therapeutic approaches (George et al., 2020). In osteosarcoma, researchers utilized CRISPR-Cas9 technology to knock out the CDK11 gene, significantly reducing the proliferation and invasion capabilities of the cells, and thereby validating the gene’s potential as a therapeutic target for osteosarcoma (Feng et al., 2015). Furthermore, the development of CRISPR-Cas9 technology has propelled advances in genetic screening, particularly in the modification of CHO host cells. CHO cells, as the preferred choice for the production of exogenous antibody-based drugs, are crucial for therapeutic efficacy in terms of the quality and quantity of antibodies expressed. Ronda et al. were the first to apply CRISPR/Cas9 technology to modify CHO cells by targeting the knockdown of two genes that affect antibody glycosylation modification, thereby optimizing the biological activity of the antibody (Ronda et al., 2014).

The capabilities of CRISPR/Cas9-mediated genome editing extend beyond simple knockout or knock-in within cells; they also allow for a deeper exploration of the biological mechanisms of cancer, including oncogene addiction, drug target assessment, and modeling of drug resistance.

##### 2.3.1.1 Validating oncogene addiction

CRISPR/Cas9 technology enables researchers to tag chemically regulated degradation determinants onto endogenous genes, facilitating precise control over specific target genes. This method allows scientists to place a target gene under the regulation of a chemical switch and evaluate the gene’s dependency status in cancer cells by monitoring the growth and survival of these cells upon the addition or removal of a specific chemical inducer. For instance, in studies investigating the RNA splicing factor SF3B1 as a potential drug target, CRISPR/Cas9 technology was employed to label the wild-type (WT) and mutant alleles of SF3B1 separately (Fernandez et al., 2024). It was observed that in two cancer cell lines harboring SF3B1 mutations, the deletion of the mutant allele did not impact cell proliferation, whereas the deletion of the wild-type allele proved lethal. This finding challenges the conventional view that cancer cells become reliant on mutant oncogenes and suggests that potential SF3B1 inhibitors must preserve the wild-type protein to avoid inducing significant targeting toxicity in normal cells.

##### 2.3.1.2 Evaluating drug targets and modeling drug resistance

CRISPR/Cas9 technology can be utilized to accurately model oncogenic chromosomal translocation events, which are crucial in various cancer types. By designing specific sgRNA pairs that target particular chromosomal translocation sites, researchers can efficiently reconstruct these events in human cell lines, thereby providing a powerful tool for drug target validation and studies on drug resistance. For instance, chromosomal translocations such as EML4-ALK, KIF5B-RET, and CD74-ROS1 (Jiang et al., 2021; Yeung et al., 2022), which are present in lung adenocarcinoma, as well as the EWSR1-FLI1 translocation in Ewing sarcoma and the RUNX1-ETO translocation in acute myelogenous leukemia (Cervera et al., 2021), have been successfully reconstructed in cellular models using CRISPR/Cas9 technology.

##### 2.3.1.3 Creation of homozygous cancer cell lines

Finally, CRISPR/Cas9 technology has been utilized to create homozygous cancer cell lines with precisely defined combinations of genetic lesions. For instance, in a study focusing on human bone marrow malignancies, researchers transduced a set of sgRNAs targeting genes that are frequently absent in direct mouse homologs into mouse hematopoietic stem cells. Following a subsequent *in vivo* selection process, leukemic clonal products containing multiple gene deletion events were successfully generated. These cell lines offer an improved model system for elucidating the functional interactions between oncogenes and tumor suppressors (Zhang G. et al., 2023).

In summary, the application of CRISPR/Cas9 technology in cancer research extends beyond high-throughput screening; it is also extensively involved in various aspects such as the validation of oncogene addiction, evaluation of drug targets, modeling of drug resistance, and the construction of complex genetically altered cell lines. With the ongoing development and refinement of this technology, CRISPR/Cas9 is anticipated to play an increasingly significant role in cancer research and treatment.

#### 2.3.2 Organoid models

Due to the scarcity of effective *in situ* models, certain cancers cannot be readily studied *in vivo*. While genetically engineered mouse models offer a potential avenue for research, they are limited by their genetic diversity and can be prohibitively expensive. Patient-derived xenografts, although useful, are not ideal for investigating tumorigenesis and drug screening. Furthermore, despite their simplicity, cancer cell lines are unsuitable for studies focused on cell differentiation, cancer stem cells, and the interactions within the tumor microenvironment. In this context, *in vitro* organoid cultures for tumor modeling represent a recent advancement that is increasingly employed in drug discovery. Organoids are micro-organs cultivated *in vitro* from adult stem cells or tissues derived from them, possessing a three-dimensional structure that can be developed to closely resemble the *in vivo* anatomy and physiology of a complete organ. These organoid cultures provide a valuable platform for studying human development and disease processes (Marciano et al., 2022). For example, the integration of patient-derived organoids (PDOs) with CRISPR screening is advancing precision medicine. A pancreatic cancer organoid biobank (n = 136) combined with a genome-wide CRISPR screen established by Tiriac et al. revealed a strong correlation between stromal density and MEK inhibitor sensitivity (r = 0.81). By editing FAP + fibroblasts *in situ*, the investigators succeeded in increasing gemcitabine efficacy by 4.2-fold, and this stromal remodeling strategy has entered phase I clinical trials.

Brain tumors are among the deadliest and most devastating forms of cancer, however, research in this area has been hindered by genetic heterogeneity and a lack of suitable models. Bian’s team has established a novel brain tumor organoid (neoCOR) model, which recapitulates brain tumor development by introducing oncogenic mutations into the organoids using transposon and CRISPR-Cas9 technologies. This new model complements existing approaches for basic and preclinical research in brain tumor biology.

To explore the etiology of breast cancer, Dekker et al. employed Cas9 to knock down four genes associated with breast cancer: P53, Pten, RB1, and NF1, in breast progenitor cells. The resulting mutated tumor-like organoids were subsequently transplanted into mice, revealing that these altered mammary organoids could be cultured for extended periods, formed estrogen receptor-positive intratumoral tumors, and exhibited sensitivity to chemotherapeutic agents. The development of these organoid models with tumor characteristics opens new avenues for further research in tumor biology (Zhao et al., 2021).

Additionally, the researchers conducted a CRISPR screen using human intestinal organoids to identify genes associated with TGF-β resistance. They successfully mitigated the noise arising from heterogeneous growth rates by employing a single-class organoid sequencing analysis method for the CRISPR screen (Zhen et al., 2022). Another study developed an optimized protocol for applying CRISPR-Cas9 screening to a 3D colorectal cancer organoid system, showcasing the versatility of CRISPR library screening in organoid models (Chen et al., 2021).

These studies not only highlight the potential of organoid models in cancer research but also offer innovative perspectives for the future development of tumor drug therapies.

#### 2.3.3 Animal models

As drug regulatory authorities in various countries impose increasingly stringent requirements on pharmacology-toxicology experiments for non-clinical studies of drugs in new drug submissions, the rapid preparation of animal models has emerged as a critical bottleneck in the successful implementation of these studies. Traditional methods for constructing mouse models rely on homologous recombination, which necessitates antibiotic screening due to its inefficient recombination efficiency and requires the use of embryonic stem cells as an intermediary. This not only prolongs the construction time but also complicates the process. For instance, in the absence of suitable embryonic stem cells, generating a knockout mouse model (e.g., C57BL/6) involves knocking out the target gene in a model mouse and subsequently backcrossing it to the target mouse strain for at least 10 generations to achieve a knockout mouse model with the desired genetic background, a process that can take several years.

In contrast, CRISPR/Cas9 technology significantly streamlines this process by enabling the direct injection of fertilized eggs, which reduces the time required to develop animal models to just a few months due to its highly efficient editing capabilities (Zhang, 2021). Since its introduction, this technology has been widely adopted for constructing knockout mouse models and has become an indispensable tool in drug discovery and development, owing to its rapidity, efficiency, and capacity to knockout and edit multiple genes simultaneously. For pharmacotoxicological studies and efficient drug screening, the incorporation of the CRISPR/Cas9 system into mouse fertilized eggs via RNA injection proves to be more effective than DNA injection, as it can generate targeted mutations in mouse embryos without being constrained by the mouse genetic strain.

CRISPR/Cas9 technology extends beyond the construction of germline mutations, it can also be employed to model a variety of human diseases by inducing the formation of *in situ* tumor models through the introduction of plasmids into animal subjects. To date, researchers have successfully established cancer models, including human liver cancer and lung adenocarcinoma, as well as various mouse disease models such as hemophilia B and heart failure, utilizing the CRISPR/Cas9 system (Ding et al., 2023).

In the realm of *in vivo* CRISPR screening, Chen et al. illustrated the significant potential of CRISPR technology. They initially developed a mouse genome-wide sgRNA library, termed mGeCKOa, which encompasses 67,405 sgRNAs targeting 20,661 protein-coding genes and 1,175 miRNA precursors. This library was subsequently integrated into a mouse non-small cell lung cancer (NSCLC) cell line, resulting in the creation of a Cas9-GFP-KPD genome-wide CRISPR screening system suitable for genetic screening across various cell lines and genetic backgrounds. To investigate tumor evolution *in vivo*, researchers employed different delivery methods (e.g., intravenous injection or *in situ* transplantation) to pinpoint genes implicated in tumor extravasation and vascularization. More comprehensive insights into tumor evolution were garnered by analyzing tumor samples collected from different time points and locations (e.g., circulating tumor cells or migrated tumors) (Song et al., 2017).

The *in vivo* CRISPR screening model can be adapted to meet experimental needs, such as in combination with drug therapy or immunotherapy, to identify genes associated with function and resistance. The implementation of Cas9-mediated activation as a function-acquisition screening strategy facilitates the identification of regulators, including proto-oncogenes, these are involved in tumor migration, thereby offering the potential to discover new therapeutic targets (Manjón et al., 2022). To enhance the accuracy of targeting key genes, researchers have proposed a sub-library screening strategy based on integrated genomic analysis, regulatory pathways, and clinical trial information for predictive purposes, which helps to reduce library size and improve screening accuracy.

In conclusion, CRISPR/Cas9 technology holds significant potential for application and research value *in vivo* screening, providing a powerful tool for elucidating critical issues such as tumor migration, while also establishing a solid foundation for drug development and personalized medicine.

## 3 Application of CRISPR-Cas9 in anticancer drug development

Screening and characterization of drug targets are essential components of new drug development, although they represent a costly endeavor. Consequently, an effective platform for the discovery and validation of drug targets is necessary. Initially, this platform relied on the genetic knockout model, which allows for the disruption of individual genes on chromosomes; however, it does not facilitate the assessment of dose-response effects. The advent of RNA interference technology (RNAi) has significantly reduced the screening time for drug targets and lead compounds (Traber and Yu, 2023). This technology effectively and selectively targets the mRNA of specific genes, thereby inhibiting their expression. Despite its advantages, RNAi technology is plagued by issues such as poor reproducibility and substantial off-target effects, which can occasionally result in erroneous conclusions. In contrast, CRISPR/Cas9 has emerged as a powerful gene editing tool, enabling the screening and editing of functional genes, validation of drug targets, and identification of small molecule drugs. This advancement provides a solid foundation for the research and development of new drugs or lead compounds (Simpson and Chuong, 2023).

### 3.1 Application in drug target identification

Most tumor characteristics are associated with genetic alterations, including the activation of oncogenes, inactivation of tumor suppressor genes, and other epigenetic changes. These alterations can enhance the proliferation and invasiveness of tumor cells, but they also present potential targets for specifically eliminating these cells. Although the Human Genome Project has been completed for some time, the functions of many genes remain inadequately understood, hindering the identification of suitable medicinal targets for certain diseases. Additionally, the challenges of large-scale genomic manipulation have constrained the understanding of disease mechanisms and the screening of drug targets. However, the advent of CRISPR/Cas9 technology offers the potential for extensive genomic manipulation, enabling the screening of drug targets through large-scale targeted editing. This technology allows for the gain or loss of function of specific genes, thereby elucidating their physiological roles and ultimately facilitating the identification of drug targets (Vaghari-Tabari et al., 2022).

#### 3.1.1 Functional gene screening

When utilizing libraries of gRNAs, CRISPR-Cas9 serves as a powerful tool for functional gene screening to identify genes that play critical roles in phenotypic expression (Su et al., 2024). The two primary high-throughput screening methods employed for identifying regulators of specific biological processes through gene function modification are loss-of-function screens and gain-of-function screens. In loss-of-function screens, gene function is diminished by inducing gene inactivation or reducing expression levels, conversely, gain-of-function screens involve the enhancement of gene expression to ascertain the corresponding phenotype. Currently, there are two types of CRISPR-Cas9 libraries available for loss-of-function screening: CRISPR knockout (CRISPRko) libraries and CRISPR-based interference (CRISPRi) libraries. According to the principles of CRISPR-Cas9, gRNA libraries targeting specific genes are delivered via a lentiviral system, which introduces gRNAs and the Cas9 nuclease into cells, resulting in specific gene knockout. Following gene editing, the mutagenized cell population can be screened for specific phenotypes, such as cell survival, proliferation, or drug resistance (Petazzi et al., 2022). In a negative screening approach, the majority of cells survive under defined selection conditions, and the gRNAs that are reduced in abundance or lost within the cells are identified through second-generation sequencing. In contrast, a positive screen is characterized by the majority of cells dying under specific screening conditions, followed by the identification of which gRNAs are enriched in the surviving cells.

In 2013, scientists at the Sanger Institute in the UK utilized CRISPR/Cas9 technology to develop a comprehensive library of sgRNAs capable of targeting all genes within the mouse genome. They successfully screened 27 known resistance genes alongside four previously unreported resistance genes. This accomplishment highlights the significant potential of CRISPR/Cas9 technology in elucidating disease mechanisms and identifying drug targets.

#### 3.1.2 Functional screening of non-coding RNA genes

With the advancement of genome-wide analysis technologies, the role of non-coding mutations in tumorigenesis has gradually gained recognition. Mutations in non-coding regions of the genome can drive cancer by altering gene expression, transcription, post-transcriptional regulation, epigenetic regulation, regulatory elements, chromatin structure, and non-coding RNAs. Non-coding elements can directly or indirectly influence the expression of oncogenes and tumor suppressor genes. However, the conventional CRISPR-Cas9 system has limitations in regulating the function of non-coding elements, particularly long non-coding RNAs (lncRNAs) (Zheng et al., 2023).

To enhance the capability of the CRISPR-Cas9 system in regulating non-coding RNAs, researchers proposed a high-throughput genomic deletion method utilizing paired guide RNA (pgRNA) libraries in 2016. The pgRNA strategy targets two cleavage sites of Cas9 proteins, with gaps of up to 23 kb, and increasing the number of sgRNA pairs can improve targeting efficiency. This strategy has demonstrated greater specificity and efficiency compared to individual CRISPR-Cas9 knockdowns, providing an effective tool for studying genome-wide lncRNAs (Arnan et al., 2022). However, targets must be designed carefully to avoid overlaps with other functional non-coding elements in the genome, such as enhancers and miRNAs, or the disruption of introns in other coding genes. Additionally, while pgRNA library methods are capable of detecting loss of function, they are complex to design and implement and are not suitable for gain-of-function screens.

In contrast, CRISPR interference (CRISPRi) and CRISPR activation (CRISPRa) methods are more effective in disrupting or stimulating long non-coding RNA (lncRNA) expression. The use of dCas9 proteins, along with repressive or activating structural domains, allows for the regulation of gene expression, including the levels of lncRNAs (Omachi and Miner, 2022). For instance, Zhang’s group combined the dCas9-VP64 protein with the MS2-p65-HSF1 fusion protein to create a SAM complex that upregulates both coding genes and intragenic non-coding RNAs. A screen conducted on the malignant melanoma cell line A375, using the BRAF inhibitor vemurafenib, identified 16 novel target sgRNAs, among which the EMICERI candidate activates neighboring genes.

In addition to non-coding RNAs, regulatory elements at the epigenomic level are also crucial for oncogene regulation. The CRISPR-Cas9-based epigenomic regulatory element screen (CERES) employs dCas9 KRAB and dCas9 p300 proteins to inhibit or activate DNAzyme I hypersensitivity sites (DHS) via sgRNA libraries. Although it was shown that gRNAs typically do not induce more than a two-fold change in gene expression, they validate a modest regulatory effect through further experimentation (Kolanu, 2024). A similar screening approach utilizing CRISPR-dCas9 KRAB inhibition was reported by Fulco et al., who applied gRNA targets across the entire genome, revealing a complex relationship between genes and enhancers. In late 2016, Fulco et al. further demonstrated that CRISPR/Cas technology can be employed to identify key regulatory elements of disease-related genes. They analyzed sequences surrounding specific genes using libraries containing a large number of sgRNA sequences, identifying non-coding regulatory elements that influence gene expression and drug resistance. These studies not only expand the application of CRISPR/Cas9 technology in non-coding genome research but also provide new insights and methodologies for disease treatment and drug development (Parsi et al., 2017).

In conclusion, complex transcriptional networks and non-coding regulatory elements associated with specific or arbitrary genes can be effectively mapped and illustrated through the customized design of sgRNA libraries.

The application of CRISPR-Cas9 technology in non-coding genome research not only enhances our understanding of gene function but also introduces innovative ideas and methods for disease treatment and drug development. Utilizing high-throughput screening and functional gene screening platforms, CRISPR-Cas9 technology is capable of elucidating the physiological functions of genes, identifying the roles of disease-related genes and non-coding RNAs, and providing a robust tool for drug target screening.

Nevertheless, CRISPR-Cas9 technology has shown high efficiency in drug target identification, but there are still some limitations in its application (Shi et al., 2015). First, CRISPR screening may produce partially functional in-frame mutants, leading to insignificant phenotypes and affecting the accuracy of screening results. Second, the applicability of CRISPR screening in non-coding regions still needs to be further validated, especially in long non-coding RNAs (lncRNAs) and enhancer regions, and it is still a challenge to design effective sgRNAs. These limitations restrict the wide application of CRISPR screening in non-coding genome research (Doench et al., 2016).

### 3.2 Application in drug target screening and validation

In the process of new drug development, the validation of drug-target interactions is a critical component (Tanoli et al., 2025). To effectively validate the target, scientists often employ the method of introducing drug-resistant mutations, which is considered a key step in identifying the target. The advent of CRISPR/Cas9 technology, in conjunction with whole genome sequencing and drug-resistant mutation screening, has significantly enhanced the efficiency and accuracy of target validation.

#### 3.2.1 Gene knockdown for target validation

CRISPR-Cas9 technology has emerged as an indispensable tool for drug target screening and validation, owing to its precise gene editing capabilities. By employing CRISPR-mediated gene knockdown, researchers can selectively remove target genes in cellular or animal models, thereby directly validating the effectiveness of specific genes or single nucleotide polymorphisms (SNPs) as drug targets (Chen et al., 2023). This allows for the assessment of the impact of gene loss on phenotypic outcomes. Such an approach not only confirms the validity of a potential drug target but also elucidates its specific mechanism of action within the disease process. For instance, in cancer research, CRISPR-Cas9 library screening has been extensively utilized to identify key genes that drive tumor growth and to evaluate the feasibility of these genes as therapeutic targets. Wang et al. validated the roles of genes such as MSH2, MSH6, MLH1, and PMS2 in DNA damage repair, as well as the resistance of the TOP2A gene to etoposide toxicity, by screening a library containing 73,000 sgRNA sequences. Conversely, Shalem et al. established a GeCKO library targeting 18,080 genes, identifying those essential for cellular growth through both positive and negative selective screening. This work also uncovered multiple genes associated with vilofenib resistance in the A375 melanoma model.

#### 3.2.2 Base editing for target validation

Recently, a novel base editing tool has been developed that activates the inducible cytidine deaminase (AID) through the recruitment of the dCas9 enzyme to specific sites (Hess et al., 2016). This results in precise point mutations without insertions or deletions (indels). This approach restricts the mutation to a 5-base window near the sgRNA target site, where AID converts cytosine (C) to uracil (U) via deamination, followed by a DNA repair mechanism that completes the final base substitution. Using this technique, Neggers et al. introduced a C528S point mutation in the XPO1 gene of acute T-cell leukemia cells, confirming that the small molecular inhibitor selinexor could specifically target and block the function of the nuclear export protein receptor XPO1.

Furthermore, the dCas9-AIDx system was employed to introduce the imatinib resistance-associated BCR-ABL mutation in K562 cells, isolating drug-resistant cells that contained the classical T315I substitution (Ma et al., 2016). These advances not only demonstrate the potential of base editing technology for the precise introduction of functional mutations but also provide powerful new tools for drug target validation. By creating new protein variants through a systematic mutagenesis approach, base editing aids in identifying functional target variants that exhibit altered drug responses, thereby elucidating drug-target relationships and modifiers for studying drug responses.

#### 3.2.3 Target validation by generating functional resistance alleles

Target validation through the generation of functional drug-resistant alleles represents a cutting-edge and highly efficient strategy in scientific research, particularly in the realm of drug discovery and development. The CRISPR/Cas9 system is employed to precisely introduce specific mutations in target genes that confer cellular resistance to particular drugs. These modified genes, referred to as functional drug resistance alleles, can mimic the mutation state of a drug’s target of action, thereby assisting researchers in validating the interaction between the drug and its target. In the context of target validation, the introduction of functional drug-resistant alleles enables cells to continue growing and proliferating in the presence of the drug, while wild-type cells are inhibited. By comparing the phenotypic differences between drug-resistant and sensitive cells under drug treatment, researchers can elucidate the mechanisms of drug-target interactions and understand how drug-resistant mutations influence drug efficacy. Additionally, functional drug-resistant alleles can be utilized to evaluate drug resistance and sensitivity, providing crucial insights for the clinical application of drugs.

For instance, in the validation of the antiproliferative drug triptolide, Smurnyy et al. from the University of Cambridge successfully identified ERCC3/XPB as its biologically relevant target using CRISPR/Cas9 technology and drug-resistant clone sequencing. They engineered multiple point mutations in the ERCC3 gene of HCT116 cells using oligonucleotide donor templates via a homology-directed repair (HDR) mechanism, which conferred resistance to tretinoin in these cells. Subsequent analysis revealed that ectopic expression of the wild-type ERCC3 cDNA in the mutant cell lines reversed the resistance phenotype, thereby demonstrating the effectiveness and specificity of the engineered point mutations.

In addition, Chu et al. utilized CRISPR/Cas9 technology to establish a link between the anti-tumor activity of spirochetes (rocaglates) and the inhibition of eukaryotic initiation factor 4A (eIF4A) (Chu et al., 2016). By introducing the F163L mutation into the eIF4A1 locus of NIH 3T3 cells, they generated a cell line exhibiting significant resistance to spirochetes. Silencing mutations were incorporated into repair oligonucleotides to prevent re-cleavage by Cas9, confirming that the mutant allele was generated through homology-directed repair (HDR). These studies not only validated eIF4A as a key target of Spirogyra but also demonstrated the efficacy of CRISPR/Cas9 in constructing functional drug resistance alleles.

The generation of functional drug-resistant alleles through CRISPR/Cas9 technology offers an efficient and precise method for drug target validation. This approach accelerates the process of new drug development and provides a novel tool for personalized medicine, enabling researchers to gain a deeper understanding of the molecular mechanisms of drug action while offering potential solutions to the challenge of drug resistance. The application of this method can facilitate the identification of more effective therapeutic targets and promote the advancement of precision medicine.

CRISPR/Cas9 technology has played an important role in drug target validation, but there are still some limitations in its application. For example, CRISPR-mediated gene knockdown may not be able to fully mimic the loss of function of drug targets, especially in the complex genetic context of multi-gene interactions, and its applicability still needs to be further validated (Webb et al., 2022). In the future, with the continuous development of the technology, CRISPR/Cas9 technology is expected to more comprehensively assess the functions of drug targets and their roles in multi-gene interactions by combining combinatorial genetic screening and multi-omics analysis (Chen and Zhang, 2018). In addition, the development of more efficient knockout tools and optimization of screening strategies will further improve the accuracy and efficiency of target validation and promote its wider application in cancer drug discovery and development (Liu et al., 2018).

### 3.3 Combinatorial genetic screening

In drug development, genome-wide association studies (GWAS) are invaluable for identifying risk-associated single nucleotide polymorphisms (SNPs). The discovery of genetic linkages between these SNPs and diseases can significantly enhance the success of drug target indications (Defo et al., 2023). However, because GWAS primarily reveal variations in non-coding sequences, assessing their functional impact poses challenges. The advent of CRISPR/Cas9 technology has facilitated the connection between these SNPs and the genes they regulate by generating isogenic cell lines, thereby expanding our understanding of the underlying biology of disease processes and aiding in the identification of drug development targets that are more likely to yield successful outcomes.

Combinatorial genetic screening approaches utilizing CRISPR-Cas9 library screening can uncover complex associations and interactions between oncogenes and metabolic functions. Multiple gene targeting systems can elucidate the potential roles of the uncharacterized transcriptome and the functions of untranslated regions. By transducing sgRNAs that target two loci within the same cell, multiple genetic modifications can be achieved. Moreover, advancements in CRISPR array coding and CombiGEM technology have facilitated the construction of multiplexed gRNA libraries, which have been tested for their effects in ovarian cancer cells, leading to the identification of two combinations of drug targets as promising therapeutic candidates (Wong et al., 2016).

CRISPR technology plays a pivotal role in constructing cancer gene-dependent maps, facilitating the identification of gene interactions (GIs) with synthetic lethality potential. These interactions represent an under-explored source of targets critical for developing drugs with broader therapeutic windows. The application of functional gene interaction (GI) mapping has successfully mapped over 21,000 pairs of drug targets through numerous parallel double-knockout assays, identifying corresponding lethal drug combinations. This systematic GI network has enabled the advancement of more personalized targeted therapies. A research team at the Broad Institute in the U.S. reported an innovative approach to simultaneously edit two independent loci using two distinct Cas9 enzymes within the same transduction. This method employed machine learning to design different sgRNAs compatible with SaCas9 and transduced the dual-CRISPR system into various cell lines, achieving gene editing through different CRISPR-Cas9 protein-mediated simultaneous knockdown and overexpression. The development of these technologies offers powerful tools for drug discovery and personalized medicine.

Although combinatorial genetic screening has demonstrated high efficiency in revealing gene interactions, its complexity may lead to difficulties in interpreting the data, especially in the case of multi-gene interactions, and its applicability in different cell types still needs to be further validated. In the future, the combination of machine learning and multi-omics analysis could allow for a more comprehensive parsing of the results of combinatorial screening and reveal the complex network of gene interactions. In addition, the development of more efficient screening tools and optimization of screening strategies will further improve the accuracy and efficiency of combinatorial screening, providing more powerful tools for drug discovery and personalized medicine.

### 3.4 Screening small molecules to overcome CAR-T therapy resistance

During the development of new drugs, accurately identifying the cellular targets of candidate molecules is critical. Immunotherapy has made significant advances in treating a wide range of cancers, with CAR-T cell therapy demonstrating particular effectiveness in B-cell malignancies (Feins et al., 2019). However, the challenges of primary and acquired resistance remain significant obstacles in this field.

Small molecule inhibitors can modulate immune cells, yet their efficacy is frequently constrained by the emergence of drug resistance. To address this issue, researchers have started utilizing CRISPR-Cas9 genome-scale screening technology to conduct extensive drug sensitivity screens aimed at identifying potential drugs that may enhance CAR-T efficacy.

In studies involving cytotoxic T cells, scientists have screened over 500 compounds along with their downstream signaling pathways, employing CRISPR technology to investigate genes that influence CAR-T cytotoxicity (Song et al., 2024). They discovered that SMAC mimetics increased the sensitivity of malignant B cells to CAR-T cells, with this effect mediated through the RIPK1 signaling pathway, which encompasses programmed cell death, including necrotic apoptosis (Dufva et al., 2020).

By integrating small molecule analysis with CRISPR library screening, researchers achieved a rapid and systematic identification of potent compounds with well-defined genetic mechanisms of action (Slivka et al., 2019). This approach not only improves the efficiency of drug screening but also provides valuable insights into the mechanisms of drug action.

CRISPR/Cas9 technology has demonstrated significant advantages in the field of drug discovery, especially in small molecule drug screening and the treatment of drug-resistant cancers, providing a powerful tool for new drug discovery. However, the application of CRISPR screening in immune cells (e.g., primary T cells) is still challenging, with low editing efficiency and its long-term safety (e.g., avoidance of immune response and genomic instability) still needs to be further evaluated, especially *in vivo* applications. In the future, the combination of single-cell sequencing and spatial transcriptomics technologies will allow for a more comprehensive resolution of the results of CRISPR screening, especially for functional screening in immune cells.

### 3.5 Multimodal functional genomics integration strategies

#### 3.5.1 *In vivo* CRISPR screening technology

In recent years, *in vivo* CRISPR screening technology has become an important tool for resolving tumor heterogeneity and drug resistance mechanisms (Chen et al., 2017). In a breakthrough study published in Nature Biotechnology, Ye et al. developed a transposon-mediated *in vivo* CRISPR library delivery system (Tuba-seq), which enabled parallel functional analyses of thousands of genes in mouse lung adenocarcinoma models (Rogers et al., 2017). The method revealed differences in the contribution of different genetic variants to tumorigenesis by quantifying the frequency of tumor-initiating cells. For example, the synergistic effect of KRAS G12D mutation and TP53 deletion enhances tumor initiation capacity by 300-fold, whereas KEAP1 deletion enhances it by only 2-fold. This high-throughput quantitative screening provides a whole new dimension for targeted therapy prioritization assessment.

The Multi-Organ Metastasis Model Screening Platform (MOMA), reported by Renz et al. in Nature, takes this technology to new heights. The team constructed a CRISPR library containing 12,000 genes and systematically identified key regulatory genes for pancreatic cancer liver metastasis and brain metastasis through a combination of intravenous injection and *in situ* transplantation. Strikingly, the study found that deletion of the S100A9 gene reduced the number of metastatic foci by 83%, while its overexpression promoted vasculomimetic formation. This spatially resolved screening strategy points the way to the development of organ-specific metastasis inhibitors (Yang et al., 2014).

*In vivo*, CRISPR screening platforms such as Tuba-seq and MOMA provide a unique perspective on tumor heterogeneity and organ-specific metastasis through innovative technological strategies. Tuba-seq uses CRISPR libraries to target tumor driver genes, and in combination with barcode labeling and single-cell sequencing, it can quantitatively analyze the effects of different mutations on tumor growth at the monoclonal level. This method not only reveals the tumor’s internal structure but also the tumor’s internal structure. This approach not only reveals intra-tumor inter-clonal competition, such as the inhibitory effect of Kras mutant clones on wild-type cells in lung cancer, but also captures microenvironment-dependent differences in gene function (Rogers et al., 2017), such as Apc deletion in intestinal stem cells with phenotypes dependent on specific ecological niches. A Tuba-seq screen identified the role of S100a4 in breast cancer brain metastasis by activating blood-brain barrier-penetrating pathways (e.g., MMP9) and is dependent on lgf2 signaling in liver metastasis (Inukai et al., 2022). In colorectal cancer models, Tuba-seq further identified the ASCL2^+^ stem cell-like cell subpopulation as a key cellular subpopulation driving liver metastasis, providing molecular evidence at single-cell resolution to study tumor heterogeneity.

The MOMA platform enables real-time dynamic tracking of tumor metastatic pathways through the integration of CRISPR screening and fluorescent reporter systems. It has precisely resolved the association between genetic variants and organ tropism, e.g., Tgfbr2-deficient breast cancer cells tend to metastasize to bone and Cdkn2a-deficiency promotes lung metastasis, and it has clarified the organ-specific regulatory role of chemokines (e.g., CXCL12) in the pre-metastatic ecological niche. In addition, MOMA revealed organ differences in immune escape, e.g., Pd-1/7 deletion inhibited growth due to increased T-cell infiltration on lung metastasis but had no significant effect in liver metastasis. Mapping the tumor microenvironment at a spatial resolution of 25 μm, MOMA revealed that CXCL6+ tumor cells recruited M2 macrophages through activation of the hepatocellular JAK-STAT3 pathway, which led to the formation of a spatially specific mechanism of the immunosuppressive microenvironment (Wu et al., 2023).

It is worth noting that these platforms break through the limitations of traditional RNA-seq methods, which can only provide population-averaged static data and are difficult to simulate complex physiological environments *in vivo*, while Tuba-seq and MOMA can achieve high-throughput screening and dynamic tracking of hundreds of genes while retaining microenvironmental interactions (including immunity, angiogenesis and other factors), which can be useful for elucidating the adaptive mechanisms of tumor cells during metastasis in different organs. Tuba-seq and MOMA can achieve high-throughput screening and dynamic tracking of hundreds of genes while preserving the microenvironmental interactions (including immunogenic factors), which provides key technical support for elucidating the adaptive changes of tumor cells in the metastatic process of different organs, the molecular regulatory network and the differences in immune escape, and thus lays the theoretical foundation for developing targeted cancer treatment strategies.

#### 3.5.2 Integrated single-cell multi-omics analysis

The combination of single-cell sequencing technology with CRISPR screening (Perturb-seq) is reshaping the functional genomics research paradigm. Dixit et al. pioneered the combination of single-cell RNA sequencing with CRISPR perturbation to map the whole transcriptome response after gene knockdown in a melanoma model. This technique was able to simultaneously resolve (1) the direct regulatory networks of target genes, (2) the reprogramming of signaling pathways triggered by secondary effects, and (3) the molecular trajectories of cellular state transitions. For example, CDKN2A knockdown not only upregulates cell cycle-related genes, but also induces EMT transformation through the AP-1 pathway, a multilevel effect that is difficult to capture in traditional batch screening (Dixit et al., 2016).

The introduction of spatial transcriptomics has further expanded the dimensions of CRISPR screening. The newly developed Slide-seqV3 technology, which enables 10 μm-level spatial resolution, successfully localized the ecological niche characteristics of EGFRvIII mutant cells in a glioblastoma model. When combined with CRISPR-mediated knockdown of microenvironmental regulators, the researchers found that TGF-β signaling inhibition reduced the area of immune-rejection microregions by 67%, providing a theoretical basis for combined immunotherapy (Rodriques et al., 2019). For example, in an acute myeloid leukemia model, Dixit et al. mapped tumor heterogeneity-driven drug resistance networks by combining CRISPR screening with single-cell transcriptome/epigenome analysis (Dixit et al., 2016).

#### 3.5.3 Comparison of deep mutation scans

In the field of target discovery, CRISPR screening complements deep mutation scanning (DMS). Adams et al. introduced all possible single amino acid mutations in the BRCA1 gene, combined with PARP inhibitor sensitivity assays, to map the drug response panorama. Compared to CRISPR screening, DMS is more advantageous in resolving cis-acting elements and protein structural domains, but lacks the ability to analyze chromosomal structural variants and non-coding regulatory elements.

Compared with traditional RNAi technology, CRISPR screening demonstrates significant advantages: (1) knockdown efficiency is increased by 3-5 fold; (2) off-target rate is reduced to 1/10; and (3) non-coding regions such as lncRNAs and enhancers can be targeted. However, RNAi is still uniquely valuable for dose-effect studies and conditional knockdown, such as time-resolved shRNA screening to capture key targets during the therapeutic window (Bhinder et al., 2014).

Compared to traditional deep mutation scanning, CRISPR saturation editing technology allows systematic assessment of the impact of all possible amino acid substitutions on drug sensitivity in an endogenous genomic setting.

## 4 Application of CRISPR technology in cancer therapy

### 4.1 Cancer genome and epigenome manipulation

#### 4.1.1 Oncogenic gene knockdown

CRISPR-Cas9 technology provides a key means for resolving the function of oncogenic genes and developing targeted therapeutic strategies. Feng’s team utilized this system to silence the endogenous CDK11 gene in osteosarcoma cells, and the results showed that the knockdown of CDK11 significantly inhibited the proliferation and invasive activity of cancer cells, suggesting that it may be a new target for osteosarcoma treatment. Similarly, in breast cancer studies, the knockdown of the shcbp1 gene not only inhibited tumor cell proliferation but also induced apoptosis (Liao et al., 2018). In addition, KLHDC4 knockdown experiments in nasopharyngeal carcinoma models further demonstrated that the technique was effective in blocking cancer cell migration and suppressing malignant phenotypes (Cheng et al., 2025).

These cases show that CRISPR-Cas9 provides an efficient tool for revealing tumorigenesis mechanisms and developing novel therapeutic modalities by precisely editing oncogenic genes. From the analysis of basic mechanisms to the validation of preclinical models, its application is gradually promoting cancer treatment towards individualization and targeting.

#### 4.1.2 Repair of oncogenic gene mutations

The application of CRISPR-Cas9 technology in cancer treatment is breaking through a multi-level strategy from single-gene precision repair to multi-gene synergistic regulation, and ultimately towards clinical translational validation. Taking *in situ* repair of single-gene mutations as an example, Sayed’s team used Prime Editor (PE3 system) to target correction of KRAS G12D mutation in a pancreatic cancer model, resulting in tumor growth inhibition of 55% without significant off-target effect (Sayed et al., 2022). Targeting BRCA1-mutated breast cancer by restoring its function through homologous recombination repair (HDR) in combination with PARP inhibitors induced tumor cell apoptosis up to 70% (Nesic et al., 2024), highlighting the therapeutic potential of precision editing for high-frequency driver mutations. Further, in bladder cancer, CRISPR-Cas9 synchronously activated oncogenes such as E-cadherin, p21, and hBax to inhibit proliferation and migration while inducing apoptosis, demonstrating the feasibility of multi-targeted synergistic intervention in solid tumors. This strategy breaks through the limitation of single gene editing and provides a new paradigm for the regulation of complex tumor microenvironment. In addition, Valletta’s team successfully restored the protein expression of ASXL1 and significantly prolonged the survival rate of mice by correcting the nonsense mutation of ASXL1 in chronic myeloid leukemia (Valletta et al., 2015), which not only verified the preclinical feasibility of driving gene repair but also revealed the deep mechanism of gene editing to reverse the malignant process of tumors. These studies have progressed from precision repair, and systemic regulation to clinical validation, signaling that CRISPR technology is being transformed from a laboratory tool to a revolutionary force in cancer therapy.

#### 4.1.3 Targeting drug resistance genes

CRISPR-Cas9 technology cracks the challenge of cancer drug resistance through a multi-dimensional strategy, from accurately modeling drug-resistant mutations to systematically exploring dependent pathways, and ultimately promoting the innovation of combination therapy strategies. Base editing technology demonstrates unique advantages in this field, for example, the use of BE4max to accurately introduce the EGFR L858R mutation in non-small cell lung cancer (NSCLC), which successfully mimics the clinical (Harrison et al., 2020). For example, BE4max was used to precisely introduce the EGFR L858R mutation in non-small cell lung cancer (NSCLC), successfully mimic the clinical resistance phenotype and screen for small molecule inhibitors to reverse the resistance. This technique not only reproduces the trajectory of tumor evolution but also provides a dynamic model for targeted drug development. Genome-wide CRISPR screening reveals the survival dependence of tumor cells from a global perspective. Wenxin Qin’s team found that targeting the mitochondrial translation process (e.g., tigecycline) could inhibit the growth of hepatocellular carcinoma, but the activation of the EGFR-ERK1/2 pathway-mediated resistance, and the combination of MEK/EGFR inhibitors could enhance the therapeutic efficacy significantly (Zhou et al., 2023). Similarly, Terai’s team combined a genome-wide screening and drug combination strategy and found that a CDK7/12 inhibitor (THZ1) could synergize with an EGFR-TKI (erlotinib) to overcome drug resistance in lung cancer (Terai et al., 2018), which provides a new idea to overcome the bottleneck of targeted therapy.

In terms of drug-resistant gene-targeted intervention, CRISPR technology reshapes the therapeutic response by precisely knocking out key genes. For example, ABCB1 knockdown in ovarian cancer resulted in a 3-fold increase in doxorubicin uptake and a 60% reduction in IC50 values (Radtke et al., 2022), while FGFR4 the knockdown in hepatocellular carcinoma reversed sorafenib resistance (Gao et al., 2017). Breast cancer studies further revealed that knockdown of MAP3K1 in the context of PIK3CA mutations enhances Akt phosphorylation, leading to decreased cellular sensitivity to Akt inhibitors (Li C. et al., 2022), suggesting a complex regulation of drug resistance by a network of gene interactions. Multi-target explorations in gliomas are more representative: ATRX knockdown sensitizes temozolomide, NOTCH1 overexpression predicts poor prognosis, PCM1 deletion inhibits proliferation, and GLI1 knockdown in combination with pentafluoroalcohol induces apoptosis (Yuan et al., 2022; Wan et al., 2017; Wang et al., 2016; Han et al., 2024), systematically mapping the panorama of therapeutic resistance in gliomas. In addition, for HPV-associated cervical cancer, CRISPR targeting of the E6/E7 oncogene enhances the effect of cisplatin and radiotherapy (Ling et al., 2020), corroborating the universality of gene editing to sensitize conventional therapies.

These studies advance from molecular mechanism analysis to preclinical validation: on the one hand, drug resistance models are constructed and targets are screened by editing technology, on the other hand, drug resistance genes or synergistic signaling pathways are directly interfered with, ultimately forming a closed-loop research system of “mechanism discovery-target validation-combination strategy”. CRISPR technology is becoming a key tool to crack the drug resistance of cancer, promoting the tumor treatment to precision and dynamization.

#### 4.1.4 Epigenetic regulation

CRISPR-Cas technology is reshaping the targeting strategy of cancer therapy in all aspects from epigenetic regulation to the non-coding RNA field. In the direction of epigenetic regulation, epigenetic alterations of tumor-related genes (e.g., DNA methylation, and aberrant histone modifications) have been shown to be one of the core mechanisms driving carcinogenesis. Based on this, researchers have realized site-specific epigenetic editing by fusing epigenetic modifying enzymes or transcriptional regulatory domains through dCas9. For example, Wong’s team significantly inhibited the proliferation and growth of ovarian cancer cells by targeting the knockdown of KDM4 (histone demethylase) or BRD4 (bromodomain protein) using the CRISPR-dCas9 system (Liu et al., 2023b). Similarly, Law’s team observed decreased growth and metastatic ability of hepatocellular carcinoma (HCC) in both *in vitro* and *in vivo* experiments by knocking down the deconjugating enzyme HELLS (Helicase Lymphoid-Specific) (Law et al., 2019), which revealed the critical role of epigenetic factors in tumor progression. These studies not only validate the therapeutic potential of epigenetic editing but also promote the development of innovative drugs targeting DNA-modifying enzymes (e.g., methyltransferases, deacetylases).

In the field of non-coding RNA regulation, breakthroughs in CRISPR-Cas13 technology have further expanded the boundaries of gene editing applications. The NYU team systematically identified 46 key long-chain non-coding RNAs (e.g., MALAT1, MIR17HG) that regulate cancer progression through transcriptome-scale CRISPR-Cas13 screening, in which knockdown of MALAT1 inhibits tumor metastasis, while the regulation of MIR17HG affects cell cycle progression (Liang et al., 2024). This discovery provides a new direction for the development of RNA-targeted therapies (e.g., antisense oligonucleotides, small molecule inhibitors), and in particular, opens up a pathway for intervention in non-coding RNA targets that are traditionally “non-druggable”.

From epigenetic to non-coding RNA, CRISPR technology is gradually building a precise regulatory network for cancer treatment through multi-dimensional editing capabilities. On the one hand, dCas9-mediated epigenetic modification directly targets the genome “software layer” to reshape the epigenetic state of tumor cells; on the other hand, Cas13 intervention on non-coding RNAs regulates the gene expression network from the post-transcriptional level. The synergistic application of these two strategies is expected to break through the limitations of a single target and provide a systemic solution to overcome tumor heterogeneity and drug resistance.

### 4.2 Applications in cancer immunotherapy

**FIGURE 3:**
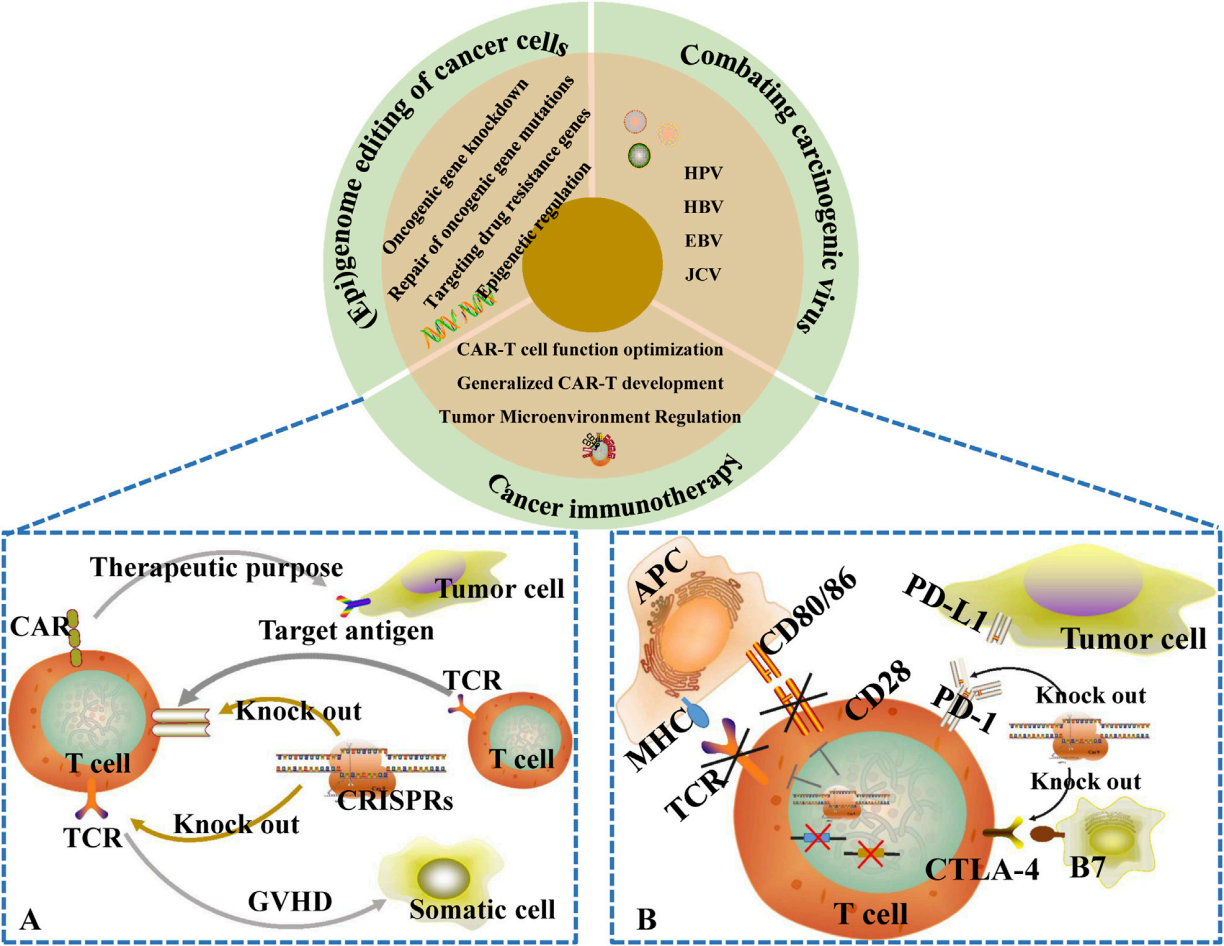
Application of CRISPR technology in cancer therapy. A normal immune T cell was genetically modified to remove the T cell endogenous αβT cell receptor gene and the human leukocyte antigen I (HLA I) class of coding genes upon introduction of the CAR sequence to prevent an anti-host reaction when used in different patients **(A)**, Knockdown of immune checkpoint-related genes, including PD-1 and CTLA-4 genes, using CRISPR/Cas9 gene editing technology can improve the effectiveness of tumor immunotherapy **(B)**.

CRISPR-Cas9 technology revolutionizes cancer immunotherapy through multidimensional strategies, from immune cell engineering modification to tumor microenvironment (TME) reprogramming, driving the therapeutic paradigm toward precision and universality. In terms of CAR-T cell function optimization, CRISPR technology significantly enhances anti-tumor activity and reduces the risk of allogeneic rejection by targeting and knocking down immune checkpoint genes (e.g., PD-1, CTLA-4) and endogenous T cell receptors (TRAC/TRBC) (Figure 3). For example, Stadtmauer’s team applied PD-1 knockdown CAR-T cells in patients with advanced non-small cell lung cancer, resulting in a 30% increase in objective remission rate and an extension of median overall survival to 42.6 weeks (NCT03044743); while CD58 knockdown reversed immune escape in lymphoma and increased CAR-T killing efficiency by 40%. By knocking down the PD-1 gene in T cells, researchers significantly improved the persistence and killing capacity of CAR-T cells in solid tumors, such as glioblastoma. Rupp et al. (2017) used Cas9-sgRNA ribonucleoprotein and exogenous single-stranded DNA templates to perform precisely targeted nucleotide substitutions at the PD-1 locus of primary T cells, thus realizing the enhancement of T cell effector function. Su et al. also showed that electrogenic plasmid-mediated PD-1 knockdown by encoding sgRNA and Cas9 is technically feasible. This approach can enhance the immune response of T cells and the cytotoxicity of cancer cell lines.

The development of generalized CAR-Ts further breaks through the limitations of autologous therapies: by knocking out HLA-I genes (e.g., B2M) and integrating targeted CAR sequences, “off-the-shelf” allogeneic CAR-T cells are constructed to survive up to two-fold longer *in vivo*, with therapeutic efficacy comparable to that of autologous cells (Ren et al., 2023). In the field of tumor microenvironment regulation, CRISPR technology has been shown to remodel the immune response by targeting immunosuppressive factors. For example, in a melanoma model, CRISPR-edited PD-L1-deficient tumor cells triggered stronger CD8^+^ T-cell infiltration and reduced tumor size by 60% in combination with anti-CTLA-4 treatment (Vaghari-Tabari et al., 2022); whereas activation of pro-inflammatory factors, such as IFN-γ expression by CRISPR, enhanced CD8^+^ T-cell infiltration and reverse the immunosuppressive state of TME. Similarly, CRISPR screening technology identifies key genes (e.g., CSF1R) that regulate macrophage polarization, providing new targets for the development of combination immunotherapies targeting tumor-associated macrophages (TAMs) (Zhao et al., 2024). In addition, CRISPR screening technology has systematically revealed the core mechanism of tumor immune escape, and a team from the University of Toronto identified 182 “core immune escape genes” in six cancer cell lines through CRISPR knockdown screening, among which autophagy-related genes (e.g., ATG12, FITM2) affect T cell killing efficiency by regulating the interferon y-signaling pathway (Ouyang et al., 2020). Knockdown of the Ptpn2 gene by CRISPR-Cas9 significantly enhances the anti-tumor efficacy of PD-1 inhibitors, a finding that provides a new idea for developing synergistic therapeutic strategies of immune checkpoint inhibitors combined with gene editing.

At the clinical translation level, several trials around the world have accelerated the implementation of CRISPR immunotherapy. A joint team at the University of Pennsylvania designed CRISPR-T cells to target sarcoma and melanoma, while Sichuan University in China focused on evaluating the efficacy of lung cancer (Bernard et al., 2022; Wang et al., 2018). These studies not only validate the safety of the technology but also open up new pathways for solid tumor treatment through the synergy of engineered T cells and microenvironmental regulation. In the future, combined with artificial intelligence-driven target prediction and novel delivery systems, CRISPR technology is expected to achieve breakthroughs from hematological tumors to solid tumors, and ultimately move towards a new era of individualized precision immunotherapy.

### 4.3 Applications in eliminating or inactivating oncogenic viral infections

CRISPR/Cas9 technology provides a precise intervention strategy for the treatment of virus-associated cancers by targeting the viral genome. The following are advances in its application to key oncogenic viruses.

#### 4.3.1 HPV-related cancers: from basic research to clinical translation

Human papillomavirus (HPV) infection is a central causative agent of cervical cancer and a variety of tumors of epithelial origin, and its oncogenicity is mainly dependent on the hijacking of the host cell cycle by E6/E7 proteins. Knockdown of the E6 or E7 genes of HPV16/18 using CRISPR/Cas9 targeting can effectively block the viral oncogenic pathway, and its anti-tumor potential has been validated in cervical cancer transformed cell lines (Inturi and Jemth, 2021). In 2017, Hu’s team further advanced to clinical translation by initiating a clinical trial of CRISPR/Cas9 in combination with TALEN targeting E6/E7 (NCT03057912) to explore its efficacy and safety against persistent HPV infection and cervical intraepithelial neoplasia, marking a critical step in moving the technology from the laboratory to the clinic. The CRISmers system developed by Wang Yu’s team screened for RNA aptamers targeting SARS-CoV-2, whose flexible structural properties are resistant to viral mutations, and may be applied to broad-spectrum therapy for cancer-associated viruses (e.g., HPV) in the future (Zhang J. et al., 2023).

#### 4.3.2 HBV and liver cancer: a breakthrough attempt to remove cccDNA

Hepatitis B virus (HBV) infection is an important causative agent of hepatocellular carcinoma (HCC), and the persistence of its covalently closed circular DNA (cccDNA) is a therapeutic difficulty. CRISPR/Cas9 can effectively destabilize the viral genome by specifically cleaving the HBV cccDNA (Seeger and Sohn, 2014), which provides a new pathway for the curative clearance of HBV infection. Although editing strategies for cccDNA are still in the preclinical development stage (Ramanan et al., 2015), their potential in HCC prevention and treatment has attracted widespread attention.

#### 4.3.3 EBV-associated tumors: precision intervention during the latent infection period

Epstein-Barr virus (EBV) is closely associated with malignant tumors such as Burkitt’s lymphoma and nasopharyngeal carcinoma, etc. CRISPR/Cas9 can inhibit viral protein expression and block oncogenic signals by targeting the genome of EBV during the latent phase (e.g., key genes such as LMP1, EBNA1, etc.) (Yang et al., 2014). For example, editing the viral genome in an EBV-positive gastric cancer model significantly inhibited tumor growth, providing a theoretical basis for antiviral-anti-cancer combination therapy.

#### 4.3.4 JCV and rare tumors: from mechanism exploration to therapeutic dawn

There is no effective treatment for progressive multifocal leukoencephalopathy (PML) caused by JC virus (JCV) infection, which is highly lethal, and CRISPR/Cas9 has successfully inhibited viral replication in a glial cell model by either cleaving the JCV genome or knocking out the genes coding for the JCV T-antigen (Wollebo et al., 2015), which provides the first potential intervention tool for PML treatment. Although an in-depth evaluation of its *in vivo* safety and delivery efficiency is still required, this breakthrough ignites hope for gene therapy for rare virus-associated tumors.

## 5 Future perspectives: challenges and prospects of CRISPR technology in cancer therapy and drug development

### 5.1 Application and challenges of CRISPR technology in personalized cancer therapy

The rapid development of CRISPR technology has opened a new path for personalized cancer therapy. By integrating genome editing, patient-derived models (e.g., tumor-like organs), and multi-omics analysis, researchers are able to more accurately model patients’ tumor characteristics and develop personalized treatment strategies. For example, the introduction of specific gene mutations in patient-derived organoids using CRISPR technology can screen for combinations of drugs that are effective in a particular patient, thereby providing customized treatment options (Takeda et al., 2019). In addition, the combination of CRISPR screening and multi-omics analysis enables researchers to identify key signaling pathways in tumor cells and develop multimodal therapeutic strategies, such as knocking out drug resistance-related genes and combining them with immune checkpoint inhibitors to enhance tumor sensitivity to immunotherapy. As the clinical translation of CRISPR technology accelerates, it is expected to provide more precise treatment options for patients by repairing oncogenic mutations or activating oncogenes in the future (Li Y. et al., 2022). However, the application of CRISPR technology in personalized cancer therapy still faces many challenges, including immune responses *in vivo* applications, genomic instability issues, and the limitation of lower editing efficiency in solid tumors. In the future, by combining multi-omics analysis and developing high-fidelity Cas9 variants, it is expected to further improve the safety and efficacy of CRISPR technology and promote its wider application in cancer therapy.

### 5.2 Challenges and prospects of CRISPR in tumor drug development

In the field of tumor drug discovery and development, the issue of protein drugability remains a major challenge. Currently, only about 20% of human proteins can be directly targeted by small molecules, which limits the scope of drug development. Traditional target-based drug screening methods, while offering advantages in structure-activity relationship optimization and biomarker development, also have limitations, resulting in many screens failing to identify suitable drug candidates. In recent years, phenotype-based drug screening methods have gradually emerged and combined with advances in robotics and imaging technologies to realize multi-parameter analysis and improve screening quality (Moffat et al., 2014).

CRISPR/Cas9 technology has become a powerful tool for drug target discovery and validation due to its high efficiency. By targeting exons encoding functional structural domains of proteins and inducing mutations, CRISPR/Cas9 technology enables large-scale screening of proteins or structural domains that are critical for cancer cell growth and survival, providing important support for identifying effective targets for drug action (Wang H. X. et al., 2017). However, CRISPR/Cas9 screening strategies also face challenges, such as the possibility of generating in-frame mutants that retain some of their functions, resulting in insignificant phenotypic differences. To address this issue, researchers generated more null mutations by mutating exons encoding functional domains of proteins, which significantly improved the efficiency of negative selection and successfully screened multiple known and potential drug targets (Wang T. et al., 2017). This approach is expected to improve the accuracy and efficiency of CRISPR/Cas9 technology in drug screening and bring new breakthroughs in the field of cancer therapy.

Despite the great potential of CRISPR/Cas9 technology in gene editing and tumor drug discovery, its application still faces technical and safety challenges, including off-target editing risks and possible unintended consequences of DNA double-strand breaks. Future research needs to be dedicated to reducing off-target effects, enhancing editing precision and efficiency, and accelerating the clinical translation of the technology. The construction of a comprehensive platform that integrates the functions of CRISPR editing, gene expression regulation and cell fate determination can more comprehensively mimic the *in vivo* environment, thus accelerating the translation process from the laboratory to the clinic (Doench et al., 2016).

### 5.3 Multidisciplinary cross-pollination to promote the clinical translation of CRISPR technology

In the future, the clinical translation of CRISPR technology can be further promoted through multidisciplinary cross-collaboration (e.g., bioinformatics, materials science and clinical medicine). For example, combining bioinformatics to optimize sgRNA design, combining materials science to develop more efficient delivery systems, and combining clinical medicine to evaluate the safety and efficacy of CRISPR technology in patients (Tsai et al., 2017). With the deepening of related research and technological innovation, CRISPR technology is expected to realize a wider application in the field of cancer treatment and provide better treatment options for patients.
